# Effects of SGLT2 inhibitors on body composition and potential sarcopenia-related outcomes in type 2 diabetes mellitus: a network meta-analysis

**DOI:** 10.3389/fendo.2026.1877008

**Published:** 2026-07-29

**Authors:** Xiaona Li, Yanrui Zhang, Luxun Wang, Yuanyuan Zhao

**Affiliations:** 1 Department of Pharmacy, Yellow River Central Hospital, Zhengzhou, China; 2 Department of Respiratory Medicine, Yellow River Central Hospital, Zhengzhou, China

**Keywords:** body composition, LEAN MASS, sarcopenia, skeletal muscle mass, sodium-glucose transporter 2 inhibitors, type 2 diabetes mellitus

## Abstract

**Background:**

Although Sodium-glucose transporter 2 (SGLT2) inhibitors provide substantial cardiovascular and renal benefits in type 2 diabetes mellitus (T2DM), their effects on sarcopenia-related body composition outcomes remain uncertain.

**Methods:**

Eight databases were searched for randomized controlled trials (RCTs) enrolling adults with T2DM that compared SGLT2 inhibitors with placebo or other glucose-lowering therapies, with a minimum follow-up of 12 weeks. A frequentist random-effects network meta-analysis was conducted. Primary outcomes were skeletal muscle mass (SMM) and lean mass (LM). Secondary outcomes included fat mass (FM), body fat percentage (PBF), visceral and subcutaneous fat, anthropometric measures, and safety outcomes.

**Results:**

Thirty-three RCTs involving 5,399 participants were included. No SGLT2 inhibitor significantly reduced SMM compared with placebo (all 95% confidence intervals crossed zero). For LM, empagliflozin (mean difference [MD] −1.22 kg, 95% CI −1.71 to −0.72), ipragliflozin (MD −0.99 kg, 95% CI −1.45 to −0.54), and canagliflozin (MD −0.80 kg, 95% CI −1.39 to −0.21) were associated with modest reductions in LM compared with placebo, whereas dapagliflozin and tofogliflozin showed neutral effects. In contrast, SGLT2 inhibitors consistently reduced FM (e.g., dapagliflozin MD −1.75 kg, 95% CI −2.63 to −0.87), PBF, visceral and subcutaneous fat, body weight (BW), body mass index (BMI), and waist circumference (WC). Safety analyses were limited by sparse networks and should be interpreted cautiously.

**Conclusion:**

In adults with T2DM, SGLT2 inhibitors reduce adiposity without clear evidence of clinically meaningful reductions in SMM. However, because muscle strength and physical performance outcomes were rarely reported, the current evidence is insufficient to determine the impact of SGLT2 inhibitors on sarcopenia risk. Findings for LM should be interpreted cautiously, as LM does not directly reflect muscle mass.

**Systematic review registration:**

https://www.crd.york.ac.uk/prospero/, identifier CRD420261379719.

## 1 Introduction

Sarcopenia is a progressive skeletal muscle disorder. It is characterized by declines in muscle mass, strength, and physical performance. These declines increase the risks of disability, hospitalization, and mortality. Recent consensus statements from the Global Leadership Initiative in Sarcopenia (GLIS) and the Asian Working Group for Sarcopenia (AWGS) emphasize these domains as core diagnostic components and highlight that reductions in muscle mass may precede functional impairment (1, 2).

Although traditionally age-related, sarcopenia is increasingly recognized as a common comorbidity of type 2 diabetes mellitus (T2DM), showing a substantially higher prevalence than in non-diabetic populations (1, 3, 4). Epidemiological studies indicate that the prevalence of sarcopenia in patients with T2DM is approximately two- to threefold higher than that in non-diabetic individuals, with reported rates reaching around 27.9% in older populations (4, 5).This association is driven by interrelated mechanisms, including insulin resistance, chronic inflammation, mitochondrial dysfunction, and advanced glycation end product accumulation, which impair muscle protein homeostasis. In turn, skeletal muscle loss further exacerbates insulin resistance, forming a bidirectional pathological cycle and contributing to adverse outcomes such as impaired physical function, increased fall risk, and higher cardiovascular and all-cause mortality (6, 7).

Sodium-glucose transporter 2 (SGLT2) inhibitors are a cornerstone therapy for T2DM, recommended as first-line or early combination agents due to their glucose-lowering efficacy and established cardiovascular and renal benefits (8, 9). SGLT2 inhibitors induce glycosuria and a negative energy balance, leading to modest but sustained weight loss (10). However, a key concern has emerged: does this weight loss come mainly from fat, or does it also reduce lean and skeletal muscle mass? The latter would potentially increase sarcopenia risk (11). Conversely, other studies have demonstrated significant reductions in adiposity without corresponding losses in muscle mass or strength, indicating a neutral effect on muscle preservation (12, 13).

Given these inconsistencies and heterogeneity in study design and outcome assessment, a comprehensive synthesis is warranted. In this study, we conducted a network meta-analysis of randomized controlled trials to compare the effects of different SGLT2 inhibitors on body composition parameters in patients with T2DM. By integrating both direct and indirect evidence, this analysis aims to provide a more precise comparative evaluation of their effects on muscle- and adiposity-related outcomes and to inform their potential implications for sarcopenia risk.

## 2 Materials and methods

This systematic review and network meta-analysis was conducted following the Cochrane Handbook for Systematic Reviews of Interventions (14). Reporting followed the PRISMA 2020 statement for systematic reviews (15)and the PRISMA extension statement for network meta-analyses (16). The study protocol was registered in PROSPERO (CRD420261379719). All prespecified outcomes were reported as planned, and no deviations from the protocol were identified during the review process.

### 2.1 Search strategy

We searched four English databases (PubMed, Web of Science, Embase, and Cochrane Central Register of Controlled Trials [CENTRAL]) and four Chinese databases (CNKI, CBM, Wanfang, VIP), from database inception to 27 January 2026. The search strategy combined controlled vocabulary terms (e.g., MeSH and Emtree) and free-text keywords related to SGLT2 inhibitors, T2DM, and sarcopenia or body composition outcomes. In addition, validated filters for randomized controlled trials (RCTs) were applied. Furthermore, the reference lists of the included studies and relevant systematic reviews were manually screened to identify additional eligible studies. The detailed search strategies for each database are provided in **Appendix 1**.

### 2.2 Eligibility criteria

#### 2.2.1 Inclusion criteria

Inclusion criteria were as follows: (1) adults (≥18 years) with T2DM, regardless of sarcopenia status; (2)a follow-up duration of at least 12 weeks; (3) randomized controlled trials (RCTs) comparing SGLT2 inhibitors against placebo or any active glucose-lowering agent (including but not limited to metformin, sulfonylureas, dipeptidyl peptidase-4 [DPP-4] inhibitors, Glucagon-Like Peptide-1 Receptor Agonist[GLP-1RA], or insulin) were eligible; (4)studies reporting at least one sarcopenia-related outcome, specifically skeletal muscle mass (SMM), lean mass (LM), skeletal muscle index (SMI), gait speed, and handgrip strength.

#### 2.2.2 Exclusion criteria

Exclusion criteria were as follows: (1) studies with incomplete or unavailable data; (2) studies that did not report the primary outcomes of interest; (3)trials with a crossover design; (4) duplicate publications; (5) animal studies.

### 2.3 Study selection

All retrieved records were exported to EndNote, and duplicate entries were removed automatically and then manually verified. The screening process was conducted in two stages. First, two independent reviewers screened the titles and abstracts of all unique records against the prespecified eligibility criteria. To maximize sensitivity, any record deemed potentially relevant by either reviewer proceeded to the next stage. Second, the same two reviewers independently assessed the full texts of the selected studies. Disagreements at either stage were resolved through discussion; if consensus could not be reached, a third senior reviewer was consulted.

### 2.4 Data extraction

A prespecified data extraction sheet was used to collect key information from each eligible trial, including study characteristics (first author, year, country, follow-up duration), participant demographics (sample size, age, sex, body mass index), intervention and controls, primary outcomes (sarcopenia-related indicators: SMM, LM, SMI, gait speed, handgrip strength), and secondary outcomes (body weight [BW], body mass index [BMI], waist circumference [WC], fat mass [FM], body fat percentage [PBF], visceral fat area [VFA], subcutaneous fat area [SFA], total body water [TBW]). SMM and LM, as reported in the original studies, were extracted and analyzed as separate outcomes and were not considered interchangeable. Conventional hypoglycemic treatment referred to usual care or investigator-directed background glucose-lowering therapy, without restriction to a specific drug or class. When background treatment was predefined, the regimen reported in the original study was used. In addition, we assessed safety outcomes, including any adverse event, serious adverse event, adverse events leading to discontinuation, and genitourinary tract infections. Data extraction was conducted independently by two reviewers, with disagreements resolved by consensus or referral to a third reviewer. All primary and secondary outcomes described above were prespecified in the study protocol and were reported as planned.

### 2.5 Quality assessment

The methodological quality of each included trial was independently appraised by two reviewers using the Cochrane Risk of Bias tool (RoB 2.0). The assessment focused on five key domains: bias arising from the randomization process, bias due to deviations from intended interventions, bias due to missing outcome data, bias in outcome measurement, and bias in selection of the reported result (17). Overall risk of bias was rated as low (all domains low risk), some concerns (at least one domain with some concerns and no high-risk domains), or high (any domain rated high risk). Disagreements were resolved by consensus or referral to a third reviewer. We used the CINeMA framework to assess the certainty of evidence for SMM and LM across six domains: within-study bias, reporting bias, indirectness, imprecision, heterogeneity, and incoherence. Transitivity was assessed by comparing potential effect modifiers across interventions, including participant characteristics, geographic distribution, and measurement methods for SMM and LM (**Appendix 8**, **Supplementary Tables 8.1**-**8.4**). Baseline body composition and detailed background antidiabetic therapy were not formally assessed because they were insufficiently or inconsistently reported (18).

### 2.6 Data analysis

#### 2.6.1 Statistical models

All statistical analyses were performed using Stata software version 18.0. A frequentist framework with random-effects models was adopted for all network meta-analyses. Multi-arm trials were included as separate study-comparison records for each eligible intervention arm. Correlations arising from shared comparator groups were appropriately accounted for within the frequentist network meta-analysis framework, thereby preserving the randomized structure of the original studies and avoiding double-counting of participants. For continuous outcomes, including SMM, LM, FM, VFA, and SFA, treatment effects were expressed as mean differences (MDs) with corresponding 95% confidence intervals (CIs). For dichotomous outcomes (e.g., adverse events), odds ratios (ORs) with 95% CIs were calculated. All continuous outcomes were extracted as absolute changes from baseline. When standard deviations (SDs) were not directly reported, they were derived from available statistics (e.g., standard errors, confidence intervals, or P values). If these data were unavailable, SDs were imputed from comparable trials reporting the same outcome. The restricted maximum likelihood (REML) method was used to estimate the between-study variance (τ²).

#### 2.6.2 Assessment of heterogeneity and inconsistency

Heterogeneity was quantified using τ².Consistency between direct and indirect evidence was evaluated using both local and global approaches. A node-splitting method was employed to assess inconsistency within specific comparisons, while a design-by-treatment interaction model was used to evaluate inconsistency across the entire network. A non-significant result (P > 0.05) was considered indicative of consistency.

#### 2.6.3 Network geometry, ranking, and publication bias

Network geometry was illustrated by network plots, where node size represented the total sample size and edge thickness reflected the number of direct comparisons. Relative treatment effects were visualized using forest plots, and league tables were constructed to present all pairwise comparisons derived from the NMA. To rank competing interventions, the surface under the cumulative ranking curve (SUCRA) and cumulative ranking probabilities were calculated. Higher SUCRA values indicated a greater probability of being the most effective treatment. For outcomes in which a reduction represented clinical benefit (e.g., FM, VFA, SFA), rankings were oriented accordingly to ensure interpretability. Small-study effects and potential publication bias were examined by visual inspection of funnel plots for each direct comparison, and formally tested using Egger’s test. A two-sided P value < 0.05 was considered statistically significant.

### 2.7 Ethics statement

Ethical approval was not required for this study because all analyses were based on previously published randomized controlled trials and no individual patient data were collected.

## 3 Results

### 3.1 Study selection and characteristics

**Figure 1:**
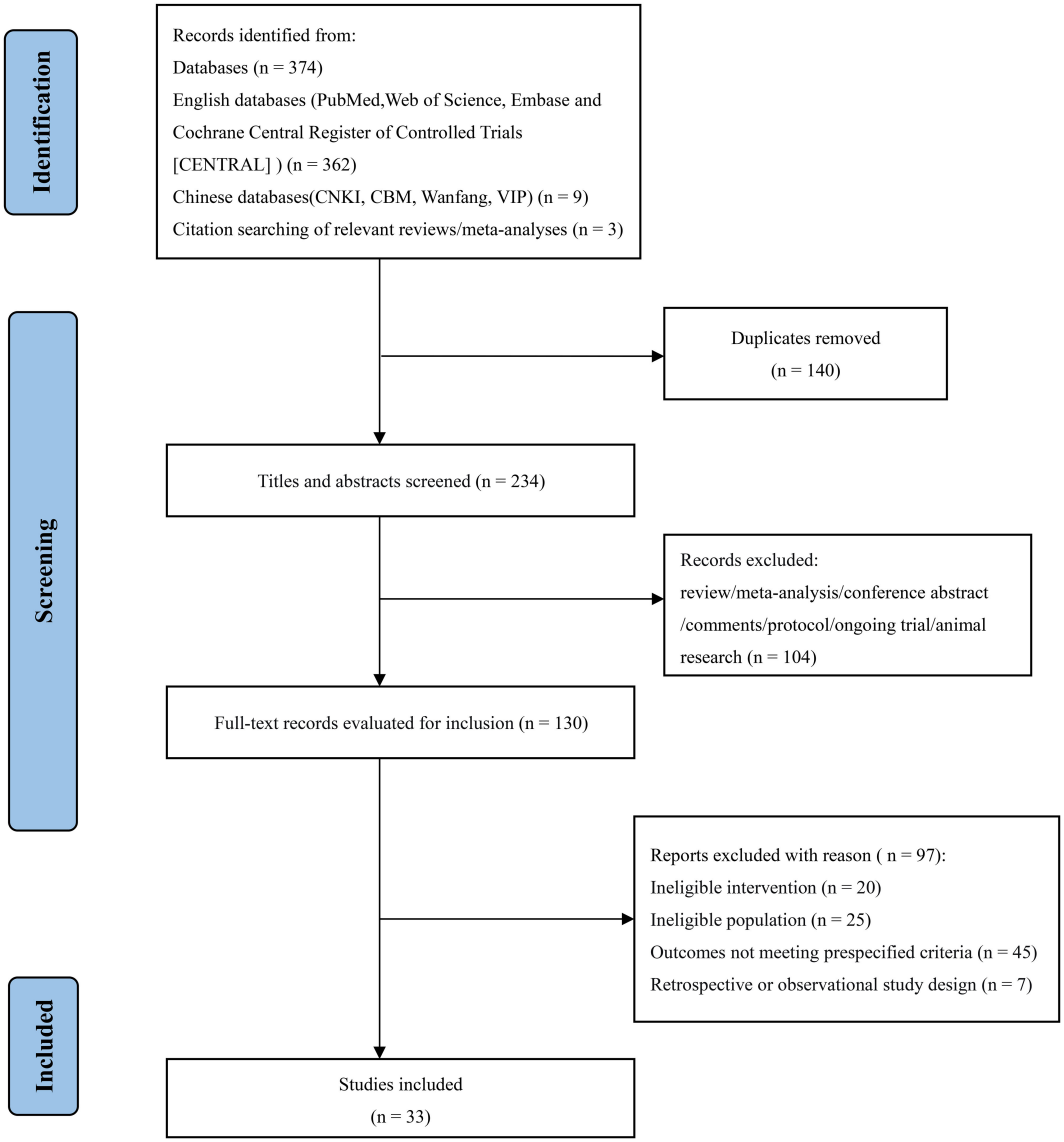
PRISMA flow diagram: summary of the search, screening, and selection process for the systematic review and meta-analysis.

A total of 374 records were identified through database searches, of which 234 remained after removal of duplicates. Following title/abstract screening and full-text assessment, 33 randomized controlled trials (RCTs) involving 5399 participants were included in the final network meta-analysis (19–51) (**Figure 1**).

The included studies were conducted in diverse clinical settings, with follow-up durations ranging from 12 to 102 weeks. The mean age of participants ranged from 46.9 to 74.2 years, and baseline BMI ranged from 22.1 to 32.8 kg/m². Body composition was assessed using dual-energy X-ray absorptiometry (DXA), bioelectrical impedance analysis (BIA) and, in a few studies, computed tomography (CT). SMM was predominantly assessed by BIA, whereas LM was primarily measured by DXA. Background hypoglycemic therapy included metformin, conventional glucose-lowering agents, insulin, sulphonylureas, DPP-4 inhibitors, and other regimens, as detailed in the supplementary table. Experimental interventions were SGLT2 inhibitors (canagliflozin, dapagliflozin, empagliflozin, ipragliflozin, tofogliflozin), with placebo or active glucose-lowering agents comparators. Detailed study characteristics are provided in **Appendix 2**.

### 3.2 Risk of bias and consistency

Risk of bias was assessed using the Cochrane Risk of Bias 2.0 tool (**Appendix 3**). Across the 33 included trials, low risk of bias was observed in most domains, including the randomization process (96.97%), missing outcome data (96.97%), outcome measurement (93.94%), and selective reporting (87.88%). In contrast, concerns were primarily identified in deviations from intended interventions (69.70%). Overall, 9 studies (27.27%) were rated as low risk, 23 (69.70%) as having some concerns, and 1 (3.03%) as high risk.

Global inconsistency tests showed no statistically significant inconsistency across the evaluated networks (all P > 0.05). Node-splitting analyses were consistent with these results, with no significant differences between direct and indirect estimates in any comparison, supporting the use of consistency in the network meta-analysis. Nevertheless, waist circumference approached statistical significance (P = 0.07), suggesting a possible trend toward local inconsistency that should be interpreted cautiously. Between-study heterogeneity was low to moderate for most outcomes (τ² ≤ 0.24), while higher heterogeneity was observed for total body water (τ² = 0.44), potentially reflecting differences in measurement methods and fluid-related variability (**Appendix 4**). Participant characteristics were generally comparable across interventions, although some variation in geographic distribution was observed (**Appendix 8**, **Supplementary Tables 8.1**, **8.2**). Measurement methods were largely consistent within outcomes, with SMM mainly assessed by BIA and LM mainly by DXA (**Appendix 8**, **Supplementary Tables 8.3**, **8.4**). These findings broadly supported the transitivity assumption, although residual indirectness could not be fully excluded because baseline body composition and detailed background antidiabetic therapy were poorly reported. Visual inspection of funnel plots did not reveal marked asymmetry for most outcomes. However, Egger’s tests suggested publication bias or small-study effects for VFA (P = 0.001), SFA (P = 0.001), WC (P = 0.039), and any adverse event (P = 0.045), whereas no evidence of publication bias was detected for the primary outcomes(SMM and LM) or most other outcomes. Several networks, particularly the SMM network, included relatively few studies, limiting the power of Egger’s test to detect small-study effects. Therefore, non-significant results should be interpreted with caution and do not exclude the possibility of publication bias. (**Appendix 9**). CINeMA assessment (**Appendix 8**) indicated moderate-to-low certainty for most comparisons; no high certainty was achieved, and indirectness was not a major concern.

### 3.3 Primary outcomes related to sarcopenia risk

#### 3.3.1 SMM

**Figure 2:**
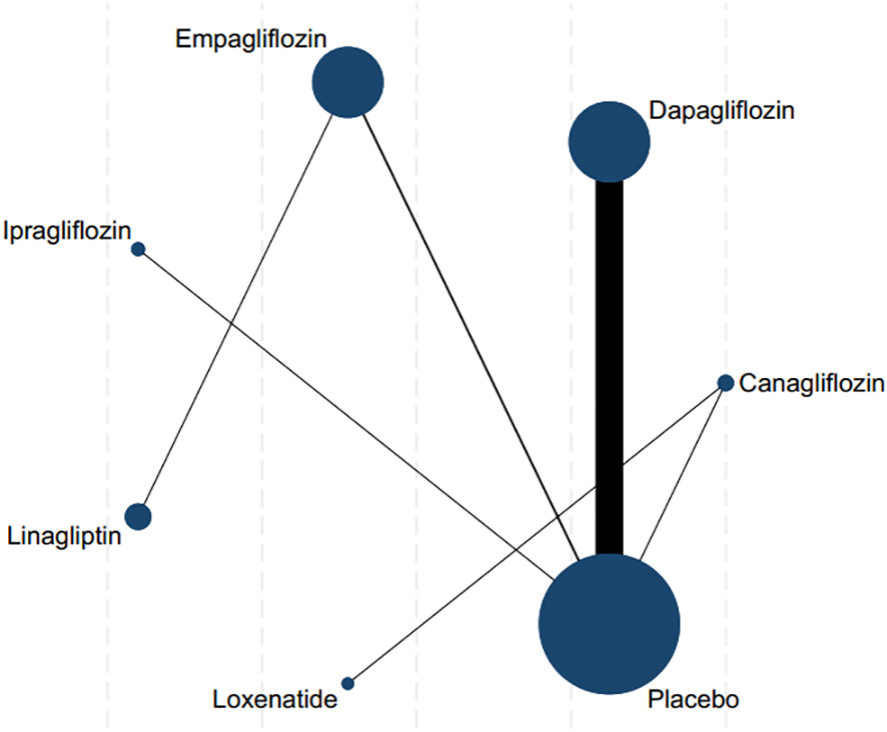
Network plot of interventions for SMM.

**Figure 3:**
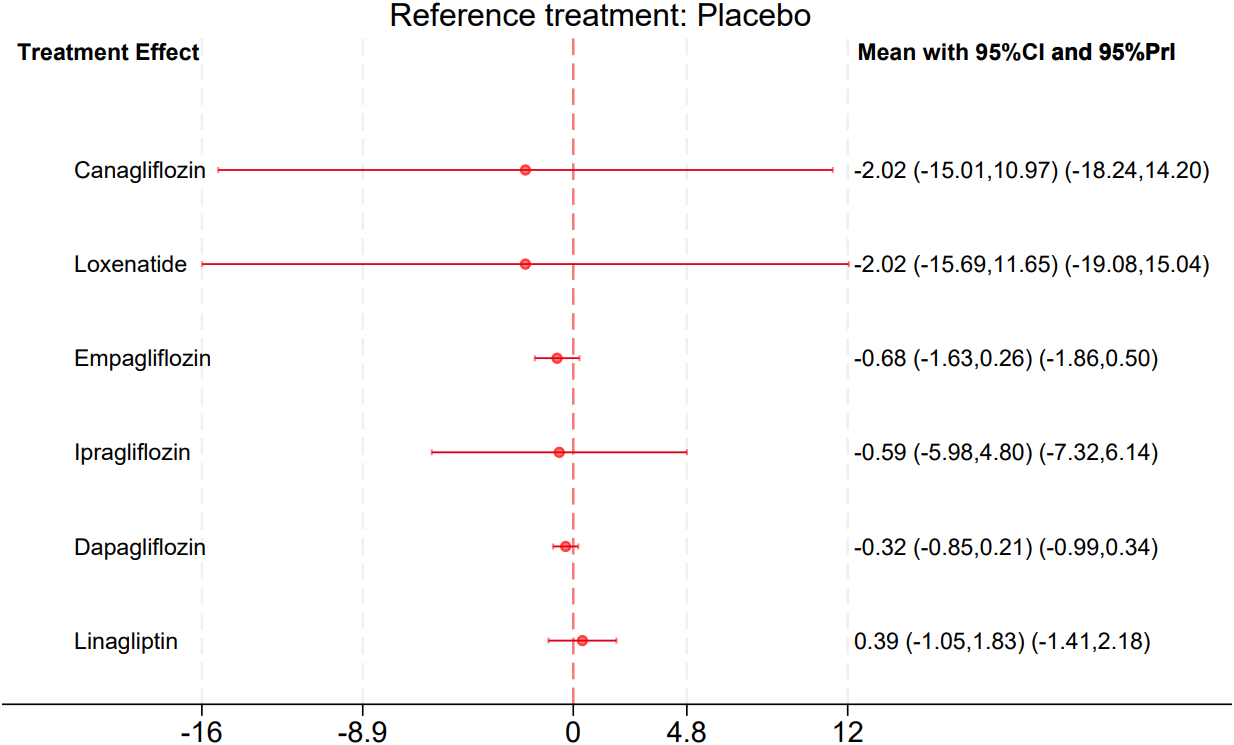
Forest plot of network effect sizes for SMM compared with placebo, negative values indicate a greater reduction in SMM, favoring placebo.

A total of 13 RCTs involving 696 participants were included in the network meta-analysis of SMM. The network plot (**Figure 2**) illustrates the evidence structure among seven active treatments and placebo. Placebo, dapagliflozin, and empagliflozin were the most extensively studied interventions, whereas ipragliflozin and loxenatide were connected to the network through a single direct comparison and contributed mainly through indirect evidence, as confirmed by the contribution matrix (**Appendix 8** **Supplementary Figure 8.5**). Compared with placebo, no SGLT2 inhibitor showed a statistically significant change in SMM. Effect estimates were small and imprecise, with 95% CIs crossing the null (**Figure 3**). The corresponding 95% prediction intervals were wide and crossed the null value for all comparisons, suggesting substantial uncertainty and heterogeneity in the treatment effects that may be observed in future studies. Pairwise comparisons between active treatments similarly showed no significant differences. SUCRA-based rankings overlapped considerably across treatments, suggesting no clear hierarchy among the evaluated agents. However, these rankings should be interpreted with caution because several interventions, particularly ipragliflozin and loxenatide, were connected to the network through a single direct comparison and therefore relied substantially on indirect evidence. Under such sparse network conditions, ranking probabilities may be unstable and sensitive to small changes in the available data (**Appendix 6**, **Supplementary Figure 6.1**, **Supplementary Table 6.1**). These findings suggest that the effect of SGLT2 inhibitors on SMM remains uncertain and, if present, is likely to be modest.

#### 3.3.2 LM

**Figure 4:**
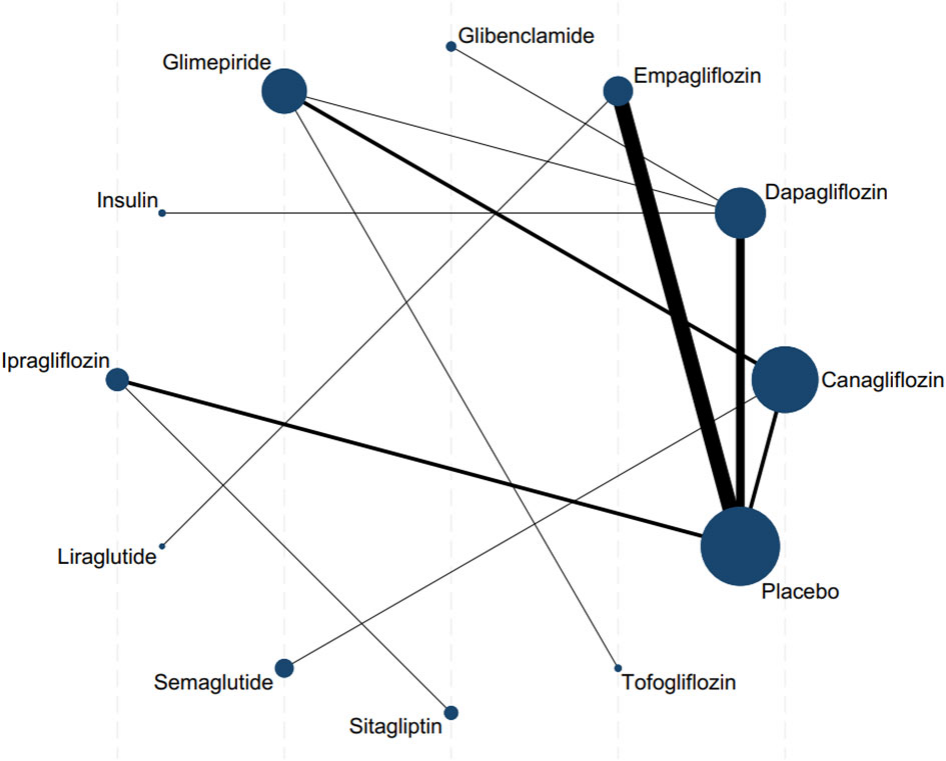
Network plot of interventions for LM.

**Figure 5:**
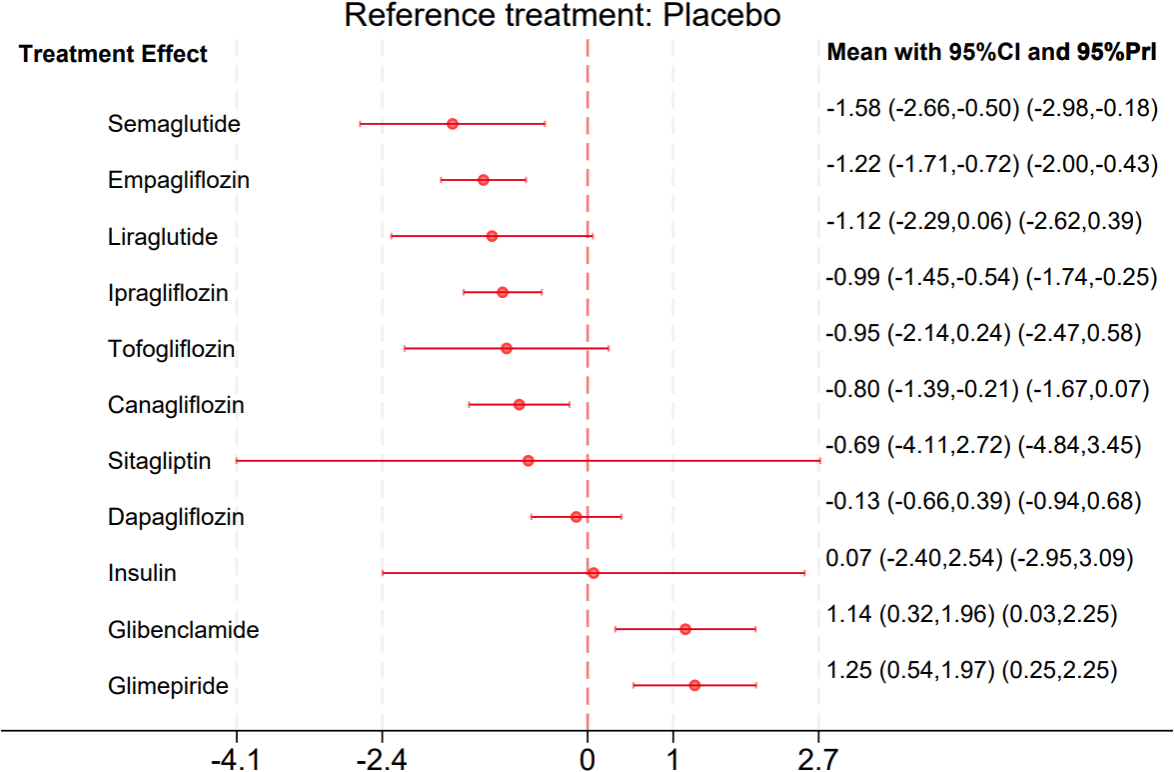
Forest plot of network effect sizes for LM compared with placebo, negative values indicate a greater reduction in LM, favoring placebo.

A total of 17 RCTs involving 1747 participants were included in the analysis of LM. The network plot (**Figure 4**) illustrates the evidence landscape for LM. Node sizes identify placebo, dapagliflozin, and canagliflozin as the most extensively studied interventions. The strongest direct evidence was observed for comparisons of empagliflozin versus placebo and dapagliflozin versus placebo, which constituted the primary evidence base within the SGLT2 inhibitor class. The LM network was more connected than the SMM network, although some active-treatment comparisons still depended largely on indirect evidence (**Appendix 8**, **Supplementary Figure 8.6**). In contrast to SMM, the effects on LM varied across agents. Compared with placebo, empagliflozin (MD −1.22, 95% CI −1.71 to −0.72) (SUCRA = 22.3%), ipragliflozin (−0.99, 95% CI −1.45 to −0.54) (SUCRA = 32.6%), and canagliflozin (−0.80, 95% CI −1.39 to −0.21) (SUCRA = 41.5%) were associated with statistically significant reductions (**Figure 5**; **Appendix 6**, **Supplementary Figure 6.2**, **Supplementary Table 6.2**). Dapagliflozin and tofogliflozin showed neutral effects, with estimates close to the null and confidence intervals crossing zero. However, most 95% prediction intervals crossed the null value (**Figure 5**), suggesting the magnitude and direction of treatment effects may vary across future clinical settings. No statistically significant differences were observed between active treatments in the network comparisons (**Appendix 7**, **Supplementary Table 7.2**). Ranking analysis further suggested variability within the class, with empagliflozin showing lower SUCRA values (22.3%) and dapagliflozin relatively higher values (64.9%), broadly consistent with the observed direction and magnitude of treatment effects. However, SUCRA values should be interpreted cautiously and do not necessarily reflect clinically meaningful differences between treatments.

### 3.4 Secondary outcomes

Consistent reductions in all adiposity-related parameters (FM, PBF, VFA, SFA, BW, BMI, and WC) were observed across SGLT2 inhibitors compared with placebo and most active comparators. The following sections summarize the key findings; detailed network estimates, league tables, and ranking results are presented in **Appendix 5–7**.

#### 3.4.1 FM and PBF

The network plots (**Appendix 5**, **Supplementary Figures 5.1**, **5.2**) illustrate the evidence landscapes for FM and PBF, with placebo, dapagliflozin, and empagliflozin representing the most extensively studied nodes. Regarding FM, SGLT2 inhibitors were generally associated with reductions in fat mass, with several agents showing statistically significant effects. Specifically, dapagliflozin (MD −1.75, 95% CI −2.63 to −0.87), ipragliflozin (−1.72, −2.86 to −0.57), canagliflozin (−1.34, −2.13 to −0.54), and empagliflozin (−1.27, −2.06 to −0.48) significantly reduced fat mass compared with placebo. Ranking analysis showed broadly consistent results, with semaglutide (SUCRA 86.0%) ranked highest overall, while dapagliflozin (SUCRA 81.0%), ipragliflozin (SUCRA 79.2%), and canagliflozin (SUCRA 67.9%) were among the higher-ranked treatments (**Appendix 6**, **Supplementary Figure 6.3**, **Supplementary Table 6.3**).

For PBF, a generally similar pattern was observed among SGLT2 inhibitors. Ipragliflozin (MD −2.08, 95% CI −3.85 to −0.31), canagliflozin (−1.93, −3.72 to −0.14), and dapagliflozin (−1.44, −2.65 to −0.24) demonstrated significant reductions compared with placebo, whereas empagliflozin (−0.87, −2.21 to 0.47) did not reach statistical significance. Ranking results were consistent with these findings, with ipragliflozin (SUCRA 89.6%), canagliflozin (83.5%), and dapagliflozin (71.5%) ranked highest(**Appendix 5**, **Supplementary Figure 5.2**; **Appendix 6**, **Supplementary Figure 6.4**, **Supplementary Table 6.4**).

#### 3.4.2 Visceral and subcutaneous fat

The evidence structures for VFA and SFA are shown in the network plots (**Appendix 5**, **Supplementary Figures 5.3**, **5.4**). In both networks, placebo, ipragliflozin, and dapagliflozin were the most extensively studied nodes, with the strongest direct evidence observed for comparisons against placebo. For VFA, only selected SGLT2 inhibitors demonstrated significant reductions versus placebo. Ipragliflozin showed the greatest reduction (MD −33.08, 95% CI −37.18 to −28.97), followed by dapagliflozin (−11.97, −20.90 to −3.04), indicating reduced visceral fat, while other treatments were not statistically significant. Ranking analysis was consistent, with ipragliflozin ranked highest (SUCRA 97.9%) (**Appendix 6**, **Supplementary Figure 6.5**, **Supplementary Table 6.5**). For SFA, dapagliflozin (MD −35.02, 95% CI −53.69 to −16.34) and ipragliflozin (−7.97, −11.61 to −4.33) significantly reduced SFA, whereas metformin increased SFA (12.17, 1.62 to 22.72). Ranking results supported these findings, with dapagliflozin ranked highest(SUCRA 99.9%), followed by ipragliflozin (SUCRA55.7%)(**Appendix 6**, **Supplementary Figure 6.6**, **Supplementary Table 6.6**).

#### 3.4.3 BW, BMI, and WC

The network plots for BW, BMI, and WC (**Appendix 5**,**Supplementary Figures 5.5**-**5.7**) showed well-connected evidence structures, with placebo serving as the central comparator and forming strong direct links particularly with canagliflozin and dapagliflozin. All evaluated SGLT2 inhibitors demonstrated significant reductions in BW compared with placebo. Canagliflozin showed the greatest reduction (MD −2.82, 95% CI −3.48 to −2.15), followed by empagliflozin (MD −2.23, 95% CI −2.80 to −1.67) and dapagliflozin (MD −2.19, 95% CI −2.80 to −1.58). Ranking analysis was consistent, with canagliflozin ranked highest (SUCRA 94.7%), followed by empagliflozin (77.8%) and dapagliflozin (75.6%).For BMI, SGLT2 inhibitors similarly demonstrated reductions, with canagliflozin showing the largest effect (MD −1.21, 95% CI −1.76 to −0.67), followed by dapagliflozin (MD −0.90, 95% CI −1.26 to −0.54)and ipragliflozin (MD −0.80, 95% CI −0.91 to −0.69). Ranking results supported these findings, with canagliflozin ranked highest (SUCRA 91.3%), followed by empagliflozin (77.9%) and dapagliflozin (73.4%).For WC, tofogliflozin showed the greatest reduction (MD −5.78, 95% CI −8.56 to −2.99), followed by canagliflozin (MD −3.82, 95% CI −5.81 to −1.82) and ipragliflozin (MD −3.70, 95% CI −4.16 to −3.24). Ranking analysis indicated that tofogliflozin (SUCRA 94.2%) and semaglutide (89.7%)were ranked highest, followed by ipragliflozin (70.4%). In contrast, sulfonylureas were associated with increases in body weight, and insulin significantly increased BMI (MD 3.80, 95% CI 1.56 to 6.04)(**Appendix 6**, **Supplementary Figures 6.7**-**6.9**, **Supplementary Tables 6.7**-**6.9**).

#### 3.4.4 TBW

The network plot for TBW (**Appendix 5**, **Supplementary Figure 5.8**) showed a simple evidence structure, with placebo as the central comparator and the strongest direct evidence observed for dapagliflozin versus placebo, while other treatments were supported by limited connections.SGLT2 inhibitors showed no statistically significant effect on TBW compared with placebo. Empagliflozin (MD −0.76, 95% CI −2.18 to 0.67) and dapagliflozin (−0.35, −1.97 to 1.28) demonstrated non-significant trends toward reduction. Ranking analysis showed no clear advantage of SGLT2 inhibitors, with linagliptin (SUCRA 67.9%) and placebo (66.3%) ranking higher than dapagliflozin (44.8%) and empagliflozin (20.9%), indicating substantial uncertainty in treatment effects(**Appendix 6**, **Supplementary Figure 6.10**, **Supplementary Table 6.10**).

### 3.5 Safety outcomes

The safety networks were relatively sparse and largely centered on placebo(**Appendix 5**, **Supplementary Figures 5.9**-**5.12**). Most effect estimates were close to unity and accompanied by wide confidence intervals, limiting the precision of comparative safety estimates. In addition, few direct comparisons between active treatments were available. Therefore, the safety findings should be considered exploratory. For any adverse event and serious adverse event, the available evidence did not indicate clear differences between treatments. For example, empagliflozin (OR 1.07, 95% CI 0.62-1.86) and dapagliflozin (OR 1.43, 95% CI 0.92-2.21) showed effect estimates close to the null, although the confidence intervals were wide. A potential signal was observed for adverse events leading to discontinuation, where dapagliflozin showed a higher point estimate (OR 2.64, 95% CI 1.00-6.96), with the lower confidence bound exactly at 1.00, indicating borderline statistical significance and substantial uncertainty. For genitourinary tract infections, no consistent evidence of increased risk was identified across SGLT2 inhibitors, although effect estimates varied between agents. Ranking analyses showed heterogeneous SUCRA distributions without a consistent hierarchy, reflecting substantial uncertainty due to limited direct comparisons and sparse network connectivity(**Appendix 5**, **Supplementary Figures 5.9**-**5.12**; **Appendix 6** for SUCRA rankings).

### 3.6 Sensitivity analyses and meta-regressions

Sensitivity analyses included the exclusion of studies with a high risk of bias and small sample sizes, restriction to BIA-based SMM and DXA-based LM measurements, exclusion of studies requiring SD imputation, and restriction of LM analyses to studies at low risk of bias. Results were generally consistent with the primary analyses, indicating that the findings were robust. (**Appendix 10**). Meta-regression analyses showed that neither measurement method nor follow-up duration category (≥24 weeks vs. <24 weeks) significantly influenced treatment effects on SMM or LM (all P>0.05; **Appendix 11**).

## 4 Discussion

### 4.1 Principal findings

In this network meta-analysis of 33 randomized trials (n = 5,399), SGLT2 inhibitors were associated with a consistent reduction in adiposity without a clear or clinically meaningful decline in skeletal muscle mass. While no individual agent significantly affected SMM, modest reductions in lean mass were observed with empagliflozin, canagliflozin, and ipragliflozin, indicating drug-level heterogeneity rather than a uniform class effect. Importantly, given that lean mass encompasses both muscle and non-muscle components, these changes do not necessarily reflect true muscle loss. In contrast, reductions in fat mass, visceral adiposity, and anthropometric indices were consistent across most agents, supporting a pattern of preferential fat loss. Nevertheless, the absence of data on muscle strength and physical performance precludes direct conclusions regarding sarcopenia risk, underscoring that these findings on body composition serve as surrogate, rather than definitive, evidence (52).

Muscle strength and physical performance are recognized as the primary diagnostic domains of sarcopenia. However, these outcomes were rarely and inconsistently reported across the included trials, making a connected network analysis infeasible. Consequently, body composition parameters were used as surrogate outcomes, as they are more consistently available and may offer clinically informative signals for early-stage risk assessment (2).

### 4.2 Comparison with previous studies and interpretation of discrepancies

Our findings refine prior evidence derived from conventional pairwise meta-analyses, which suggested that SGLT2 inhibitors reduce lean mass (-0.66 to -0.80 kg) and skeletal muscle mass (-0.35 to -0.38 kg) (53). By contrast, our network approach demonstrates that this apparent “class effect” is largely driven by specific agents, rather than reflecting a uniform pharmacological property. Notably, our finding that no individual SGLT2 inhibitor significantly reduced SMM contrasts directly with the conventional meta-analysis by Zhang et al. (2023), which reported a significant reduction in SMM with the SGLT2 inhibitor class as a whole. This discrepancy may be attributable to differences in analytical approaches (network meta-analysis vs. pairwise meta-analysis), the inclusion of indirect evidence, or the specific studies included.

This drug-level heterogeneity helps reconcile inconsistencies reported in the literature. For instance, earlier meta-analyses concluded that SGLT2 inhibitors may reduce muscle mass (54), whereas more recent long-term analyses (≥52 weeks) indicate preservation of fat-free mass despite sustained fat loss (55). Similarly, an observational study showed that empagliflozin did not significantly alter appendicular skeletal muscle index or muscle strength over 24 weeks (13),supporting the neutral SMM findings observed in our study.

Furthermore, the broader literature highlights substantial heterogeneity across studies, with reports of detrimental, neutral, and even beneficial effects on muscle mass (56). This inconsistency likely reflects differences in study populations (age, baseline sarcopenia risk), follow-up duration, measurement techniques (DXA vs BIA), and concomitant lifestyle factors, all of which influence body composition outcomes. Taken together, these findings suggest that the effects of SGLT2 inhibitors on muscle-related outcomes are context-dependent rather than uniformly detrimental.

### 4.3 Implications of GLP-1 receptor agonists and combination therapy

In the fat mass network, semaglutide ranked highest among all interventions, although this estimate was largely based on indirect comparisons and should be interpreted cautiously. This direction is consistent with the SUSTAIN 8 DXA substudy, in which semaglutide showed a numerically greater, but non-significant, reduction in total fat mass than canagliflozin (37). GLP-1 receptor-based therapies reduce fat mass, including visceral fat, but may also induce modest lean mass loss without significantly changing lean mass percentage (57, 58). In contrast, our analysis suggests that SGLT2 inhibitors reduce adiposity without clear evidence of skeletal muscle loss. These complementary profiles provide a rationale for GLP-1RA–SGLT2i combination therapy, although dedicated trials assessing body composition and muscle function are still needed.

### 4.4 Potential mechanisms underlying differential effects

Several mechanisms may underlie the observed pattern of preferential fat loss with relative preservation of muscle.

SGLT2 inhibitors induce a negative energy balance through glycosuria, promoting a metabolic shift toward lipid utilization. This fasting-like state enhances lipolysis, fatty acid oxidation, and ketogenesis (59). Experimental evidence further suggests that agents such as dapagliflozin may activate SIRT1 signaling, improve mitochondrial function, and enhance skeletal muscle insulin sensitivity (60), potentially supporting metabolic adaptation within muscle tissue.

In addition, SGLT2 inhibitors may influence muscle protein turnover. Reduced insulin levels and increased glucagon can promote mild proteolysis and gluconeogenesis, which may contribute to small reductions in LM (52). However, this effect appears limited and may be offset by improvements in systemic metabolic status, including reduced inflammation and enhanced insulin sensitivity.

Muscle-related outcomes are also likely modified by behavioral factors. Preclinical evidence indicates that resistance exercise can counteract SGLT2 inhibitor–associated muscle loss via activation of anabolic signaling pathways (e.g., AKT–mTOR) (61), suggesting that these effects are not solely drug-driven but modifiable.

Collectively, these mechanisms support a model in which SGLT2 inhibitors preferentially reduce adiposity while largely preserving muscle, rather than directly inducing sarcopenia.

### 4.5 Clinical implications

These findings have important implications for individualized diabetes management. While SGLT2 inhibitors remain a cornerstone therapy due to their established cardiovascular and renal benefits (62), our results suggest that their effects on body composition are not uniform across agents, which is particularly relevant in patients at high risk of sarcopenia.

In such populations, including older adults, frail patients, and those with low baseline muscle mass, dapagliflozin and tofogliflozin were associated with more neutral effects on lean mass in our analysis, whereas closer monitoring of muscle-related parameters may be advisable when using agents such as empagliflozin or canagliflozin.

Importantly, current sarcopenia frameworks emphasize that muscle strength and physical performance are more predictive of adverse outcomes than muscle mass alone (2). Thus, relying solely on body composition may overestimate sarcopenia risk; muscle function assessment should complement body composition evaluation in clinical practice. Management strategies should also address modifiable factors. Resistance exercise and adequate protein intake are recommended to support muscle health in patients with diabetes and may help mitigate potential muscle-related effects of glucose-lowering therapies (63).Taken together, these findings highlight the value of incorporating muscle health considerations into treatment decisions and support future guideline refinement toward more agent-specific, patient-centered approaches.

### 4.6 Strengths and limitations

This study has several strengths. The findings were robust across multiple sensitivity analyses, supporting their stability and reducing the likelihood that results were driven by small or low-quality studies. To our knowledge, this is the first network meta-analysis to compare individual SGLT2 inhibitors on sarcopenia-related outcomes, providing higher-resolution evidence than prior class-level analyses. The inclusion of 33 randomized trials, along with rigorous methodological approaches (PRISMA-NMA framework, random-effects modeling, and consistency assessment), further strengthens the reliability of the findings.

Several limitations should also be acknowledged. First, key diagnostic components of sarcopenia—muscle strength and physical performance—were insufficiently reported, and most included trials were not designed to assess sarcopenia outcomes, limiting conclusions to surrogate measures. Second, background antidiabetic therapies varied across trials, representing a key source of clinical heterogeneity. Concomitant medications may independently affect body composition, potentially confounding treatment effects and limiting comparability. Residual confounding cannot be excluded. Third, measurement methods (DXA vs. BIA) and follow-up duration (12–102 weeks) varied across studies. Although sensitivity analyses and meta-regression did not identify significant effects of either factor on SMM or LM, SMM was assessed predominantly by BIA and LM by DXA, and only a small number of studies had follow-up durations <24 weeks. Therefore, heterogeneity related to measurement methods and follow-up duration cannot be completely excluded. Fourth, most trials were short-term and industry-sponsored, limiting the generalizability of the findings. Potential publication bias was detected for several secondary outcomes, including VFA, SFA, WC, and any adverse event; therefore, these findings should be interpreted with caution because they may be influenced by small-study effects. No evidence of publication bias was observed for the primary outcomes (SMM and LM), although the possibility of publication bias cannot be completely excluded given the limited number of available studies. The minimal clinically important difference (MCID) for SMM and LM in patients with type 2 diabetes has not been established, making the clinical relevance of the observed differences uncertain. Fifth, the absence of individual patient data precluded adjustment for important confounders such as physical activity and dietary intake. Moreover, several interventions in the SMM network, particularly ipragliflozin and loxenatide, were supported by limited direct evidence, and the safety network was sparse and largely star-shaped. Consequently, treatment rankings and comparative safety estimates should be interpreted cautiously, and safety findings remain exploratory, particularly where confidence intervals were wide. Finally, baseline sarcopenia status was not routinely assessed in the included trials, and only one study excluded participants with sarcopenia at baseline. This precluded assessment of whether treatment effects differed by baseline sarcopenia status.

### 4.7 Future research

Future studies should prioritize long-term (>2 years) randomized trials incorporating muscle strength and physical performance as co-primary endpoints. Advanced imaging modalities such as MRI or CT may further improve the accuracy of muscle assessment, while individual patient data meta-analyses are needed to better identify high-risk subgroups (e.g., older adults, frail individuals, and those with low baseline muscle mass) and clarify treatment heterogeneity.

In addition, combined therapeutic strategies, such as SGLT2 inhibitors with GLP-1 receptor agonists or structured exercise interventions, may help optimize the balance between metabolic benefits and muscle preservation.

Ongoing trials evaluating functional and muscle-related outcomes—including comparisons with DPP-4 inhibitors, assessments of muscle strength and body composition across glucose-lowering therapies, studies in high-risk populations, and head-to-head comparisons between agents—are expected to address current evidence gaps and further clarify drug-specific effects on skeletal muscle (SLCTR/2025/013; KCT0010819; CTRI/2025/01/079703; KCT0010678; jRCTs041240011; ChiCTR2300074382) (64–69). These data should be incorporated into future updates of this network meta-analysis.

## 5 Conclusion

In conclusion, in adults with T2DM, SGLT2 inhibitors reduce adiposity without clear evidence of clinically meaningful reductions in SMM. However, because muscle strength and physical performance outcomes were rarely reported in the included trials, no definitive conclusion about sarcopenia risk can be drawn. Drug-level heterogeneity suggests that body composition effects are not interchangeable across agents, although differences between individual agents require further confirmation. SGLT2 inhibitors provide established cardiometabolic benefits. Their potential effects on muscle health should also be considered when choosing among individual agents.

## Data Availability

The original contributions presented in the study are included in the article/**Supplementary Material**. Further inquiries can be directed to the corresponding author/s.
