# Supplementary material for: Effects of SGLT2 inhibitors on body composition and potential sarcopenia-related outcomes in type 2 diabetes mellitus: a network meta-analysis
Source: Front Endocrinol (Lausanne). 2026 Jul 29;17:1877008. doi: 10.3389/fendo.2026.1877008 (PMC13461395; doi:10.3389/fendo.2026.1877008)
Supplement: Supplementary file 1 [file SupplementaryFile1.docx]

Supplementary Appendix

**Effects of SGLT2 Inhibitors on Body Composition and Potential Sarcopenia-Related Outcomes in Type 2 Diabetes Mellitus: A Network Meta-Analysis**

Xiaona Li^1^, Yanrui Zhang^2^, Luxun Wang^1^, Yuanyuan Zhao^1^

^1^Department of Pharmacy, Yellow River Central Hospital, Zhengzhou, China

^2^Department of Respiratory Medicine, Yellow River Central Hospital, Zhengzhou, China

Contents

[Appendix 1: Search strategy	2](#_Toc3033)

[Appendix 2: Characteristics of included studies	8](#_Toc26075)

[Appendix 3: Risk of bias of randomized clinical trials	12](#_Toc13426)

[Appendix 4: Evaluation of inconsistency and heterogeneity	15](#_Toc13027)

[Appendix 5: Network plot and forest plots of secondary outcomes	23](#_Toc22049)

[Appendix 6: SUCRA and cumulative probability plots	35](#_Toc14631)

[Appendix 7: league tables of summary estimates for SGLT2 inhibitorsderived from network meta-analysis of 33trials	63](#_Toc28799)

[Appendix 8: CINeMA Assessment and contribution matrices	77](#_Toc17222)

[Appendix 9: Funnel plots	88](#_Toc12302)

[Appendix 10: Sensitivity analyses	96](#_Toc25106)

[Appendix 11: Meta-regression analyses	100](#_Toc2525)

[Appendix 12: PRISMA-NMA Checklist	101](#_Toc9249)

Appendix 1: Search strategy

Table S1.1Search strategy of PubMed

| **#** | **Searches** |
| --- | --- |
| **1** | "Sodium-Glucose Transporter 2 Inhibitors"[Mesh] |
| **2** | ((((((((((((((((((((((((Sodium-Glucose Transporter 2 Inhibitors[Title/Abstract]) OR (Sodium-Glucose Transporter 2 Inhibitor[Title/Abstract])) OR (Sodium Glucose Transporter 2 Inhibitors[Title/Abstract])) OR (Sodium Glucose Transporter 2 Inhibitor[Title/Abstract])) OR (sodium glucose cotransporter 2 inhibitor[Title/Abstract])) OR (SGLT-2 Inhibitors[Title/Abstract])) OR (SGLT-2 Inhibitor[Title/Abstract])) OR (SGLT 2 Inhibitors[Title/Abstract])) OR (SGLT 2 Inhibitor[Title/Abstract])) OR (SGLT2 Inhibitors[Title/Abstract])) OR (SGLT2 Inhibitor[Title/Abstract])) OR (Inhibitor, SGLT-2[Title/Abstract])) OR (Inhibitor, SGLT2[Title/Abstract])) OR (Canagliflozin[Title/Abstract])) OR (dapagliflozin[Title/Abstract])) OR (empagliflozin[Title/Abstract])) OR (ertugliflozin[Title/Abstract])) OR (luseogliflozin[Title/Abstract])) OR (tofogliflozin[Title/Abstract])) OR (ipragliflozin[Title/Abstract])) OR (sotagliflozin[Title/Abstract])) OR (Henagliflozin[Title/Abstract])) OR (Remogliflozin[Title/Abstract])) OR (Enavogliflozin[Title/Abstract])) OR (Bexagliflozin[Title/Abstract]) |
| **3** | #1 OR #2 |
| **4** | "Diabetes Mellitus, Type 2"[Mesh] |
| **5** | ((((((((((((((((((((((((((((((((Diabetes Mellitus, Type 2[Title/Abstract]) OR (Diabetes Mellitus, Stable[Title/Abstract])) OR (Stable Diabetes Mellitus[Title/Abstract])) OR (Diabetes Mellitus, Noninsulin Dependent[Title/Abstract])) OR (Diabetes Mellitus, Adult-Onset[Title/Abstract])) OR (Adult-Onset Diabetes Mellitus[Title/Abstract])) OR (Diabetes Mellitus, Adult Onset[Title/Abstract])) OR (Diabetes Mellitus, Ketosis-Resistant[Title/Abstract])) OR (Diabetes Mellitus, Ketosis Resistant[Title/Abstract])) OR (Ketosis-Resistant Diabetes Mellitus[Title/Abstract])) OR (Diabetes Mellitus, Non Insulin Dependent[Title/Abstract])) OR (Diabetes Mellitus, Non-Insulin-Dependent[Title/Abstract])) OR (Non-Insulin-Dependent Diabetes Mellitus[Title/Abstract])) OR (Diabetes Mellitus, Type II[Title/Abstract])) OR (NIDDM[Title/Abstract])) OR (Diabetes Mellitus, Maturity-Onset[Title/Abstract])) OR (Diabetes Mellitus, Maturity Onset[Title/Abstract])) OR (Maturity-Onset Diabetes Mellitus[Title/Abstract])) OR (Maturity Onset Diabetes Mellitus[Title/Abstract])) OR (MODY[Title/Abstract])) OR (Diabetes Mellitus, Slow-Onset[Title/Abstract])) OR (Diabetes Mellitus, Slow Onset[Title/Abstract])) OR (Slow-Onset Diabetes Mellitus[Title/Abstract])) OR (Type 2 Diabetes Mellitus[Title/Abstract])) OR (Noninsulin-Dependent Diabetes Mellitus[Title/Abstract])) OR (Noninsulin Dependent Diabetes Mellitus[Title/Abstract])) OR (Maturity-Onset Diabetes[Title/Abstract])) OR (Diabetes, Maturity-Onset[Title/Abstract])) OR (Maturity Onset Diabetes[Title/Abstract])) OR (Type 2 Diabetes[Title/Abstract])) OR (Diabetes, Type 2[Title/Abstract])) OR (Diabetes Mellitus, Noninsulin-Dependent[Title/Abstract])) OR (T2DM[Title/Abstract]) |
| **6** | #4 OR #5 |
| **7** | "Sarcopenia"[Mesh] |
| **8** | ((((((((((((sarcopenia[Title/Abstract]) OR (Sarcopenias[Title/Abstract])) OR (muscle mass[Title/Abstract])) OR (skeletal muscle[Title/Abstract])) OR (Skeletal Muscle Mass[Title/Abstract])) OR (Muscle Tissue[Title/Abstract])) OR (Lean Mass[Title/Abstract])) OR (Hand Strength[Title/Abstract])) OR (handgrip strength[Title/Abstract])) OR (muscle strength[Title/Abstract])) OR (gait speed[Title/Abstract])) OR (muscle wasting[Title/Abstract])) OR (muscle atrophy[Title/Abstract]) |
| **9** | #7 OR #8 |
| **10** | "Randomized Controlled Trial" [Publication Type] |
| **11** | (((((randomized controlled trial[Title/Abstract]) OR (Controlled Clinical Trial[Title/Abstract])) OR (RCT[Title/Abstract])) OR (randomized[Title/Abstract])) OR (randomised[Title/Abstract])) OR (randomly[Title/Abstract]) |
| **12** | #10 OR #11 |
| **13** | #3 AND #6 AND #9 AND #12 |

**Table S1.2**Search strategy of Web of Science

| **#** | **Searches** |
| --- | --- |
| **1** | ((((((((((((((((((((((((TS=("Sodium-Glucose Transporter 2 Inhibitors")) OR TS=("Sodium-Glucose Transporter 2 Inhibitor")) OR TS=("Sodium Glucose Transporter 2 Inhibitors")) OR TS=("Sodium Glucose Transporter 2 Inhibitor")) OR TS=("sodium glucose cotransporter 2 inhibitor")) OR TS=("SGLT-2 Inhibitors")) OR TS=("SGLT-2 Inhibitor")) OR TS=("SGLT 2 Inhibitors")) OR TS=("SGLT 2 Inhibitor")) OR TS=("SGLT2 Inhibitors")) OR TS=("SGLT2 Inhibitor")) OR TS=("Inhibitor, SGLT-2")) OR TS=("Inhibitor, SGLT2")) OR TS=("Canagliflozin")) OR TS=("Dapagliflozin")) OR TS=("Empagliflozin")) OR TS=("Ertugliflozin")) OR TS=("Luseogliflozin")) OR TS=("Tofogliflozin")) OR TS=("Ipragliflozin")) OR TS=("Sotagliflozin")) OR TS=("Henagliflozin")) OR TS=("Remogliflozin")) OR TS=("Enavogliflozin")) OR TS=("bexagliflozin") |
| **2** | ((((((((((((((((((((((((((((((((TS=("Diabetes Mellitus, Type 2")) OR TS=("Diabetes Mellitus, Stable")) OR TS=("Stable Diabetes Mellitus")) OR TS=("Diabetes Mellitus, Noninsulin Dependent")) OR TS=("Diabetes Mellitus, Adult-Onset")) OR TS=("Adult-Onset Diabetes Mellitus")) OR TS=("Diabetes Mellitus, Adult Onset")) OR TS=("Diabetes Mellitus, Ketosis-Resistant")) OR TS=("Diabetes Mellitus, Ketosis Resistant")) OR TS=("Ketosis-Resistant Diabetes Mellitus")) OR TS=("Diabetes Mellitus, Non Insulin Dependent")) OR TS=("Diabetes Mellitus, Non-Insulin-Dependent")) OR TS=("Non-Insulin-Dependent Diabetes Mellitus")) OR TS=("Diabetes Mellitus, Type II")) OR TS=("NIDDM")) OR TS=("Diabetes Mellitus, Maturity-Onset")) OR TS=("Diabetes Mellitus, Maturity Onset")) OR TS=("Maturity-Onset Diabetes Mellitus")) OR TS=("Maturity Onset Diabetes Mellitus")) OR TS=("MODY")) OR TS=("Diabetes Mellitus, Slow-Onset")) OR TS=("Diabetes Mellitus, Slow Onset")) OR TS=("Slow-Onset Diabetes Mellitus")) OR TS=("Type 2 Diabetes Mellitus")) OR TS=("Noninsulin-Dependent Diabetes Mellitus")) OR TS=("Noninsulin Dependent Diabetes Mellitus")) OR TS=("Maturity-Onset Diabetes")) OR TS=("Diabetes, Maturity-Onset")) OR TS=("Maturity Onset Diabetes")) OR TS=("Type 2 Diabetes")) OR TS=("Diabetes, Type 2")) OR TS=("Diabetes Mellitus, Noninsulin-Dependent")) OR TS=("T2DM") |
| **3** | ((((((((((((TS=("sarcopenia")) OR TS=("Sarcopenias")) OR TS=(muscle mass)) OR TS=("skeletal muscle")) OR TS=("Skeletal Muscle Mass")) OR TS=("Muscle Tissue")) OR TS=("Lean Mass")) OR TS=("Hand Strength")) OR TS=("handgrip strength")) OR TS=("muscle strength")) OR TS=("gait speed")) OR TS=("muscle wasting")) OR TS=("muscle atrophy") |
| **4** | (((((TS=("randomized controlled trial")) OR TS=("Controlled Clinical Trial")) OR TS=("RCT")) OR TS=("randomized")) OR TS=("randomised")) OR TS=("randomly") |
| **5** | #1 AND #2 AND #3 AND #4 |

**Table S1.3** Search strategy of Embase

| **#** | **Searches** |
| --- | --- |
| **1** | 'sodium glucose cotransporter 2 inhibitor'/exp |
| **2** | 'sodium-glucose transporter 2 inhibitors':ab,ti OR 'sodium-glucose transporter 2 inhibitor':ab,ti OR 'sodium glucose transporter 2 inhibitors':ab,ti OR 'sodium glucose transporter 2 inhibitor':ab,ti OR 'sglt-2 inhibitors':ab,ti OR 'sglt-2 inhibitor':ab,ti OR 'sglt 2 inhibitors':ab,ti OR 'sglt 2 inhibitor':ab,ti OR 'sglt2 inhibitors':ab,ti OR 'sglt2 inhibitor':ab,ti OR 'inhibitor, sglt-2':ab,ti OR 'inhibitor, sglt2':ab,ti OR 'canagliflozin':ab,ti OR 'dapagliflozin':ab,ti OR 'empagliflozin':ab,ti OR 'ertugliflozin':ab,ti OR 'luseogliflozin':ab,ti OR 'tofogliflozin':ab,ti OR 'ipragliflozin':ab,ti OR 'sotagliflozin':ab,ti OR 'henagliflozin':ab,ti OR 'remogliflozin':ab,ti OR 'enavogliflozin':ab,ti OR 'bexagliflozin':ab,ti |
| **3** | #1 OR #2 |
| **4** | 'non insulin dependent diabetes mellitus'/exp |
| **5** | 'Diabetes Mellitus, Type 2':ab,ti OR 'Diabetes Mellitus, Stable':ab,ti OR 'Stable Diabetes Mellitus':ab,ti OR 'Diabetes Mellitus, Noninsulin Dependent':ab,ti OR 'Diabetes Mellitus, Adult-Onset':ab,ti OR 'Adult-Onset Diabetes Mellitus':ab,ti OR 'Diabetes Mellitus, Adult Onset':ab,ti OR 'Diabetes Mellitus, Ketosis-Resistant':ab,ti OR 'Diabetes Mellitus, Ketosis Resistant':ab,ti OR 'Ketosis-Resistant Diabetes Mellitus':ab,ti OR 'Diabetes Mellitus, Non Insulin Dependent':ab,ti OR 'Diabetes Mellitus, Non-Insulin-Dependent':ab,ti OR 'Non-Insulin-Dependent Diabetes Mellitus':ab,ti OR 'Diabetes Mellitus, Type II':ab,ti OR 'NIDDM':ab,ti OR 'Diabetes Mellitus, Maturity-Onset':ab,ti OR 'Diabetes Mellitus, Maturity Onset':ab,ti OR 'Maturity-Onset Diabetes Mellitus':ab,ti OR 'Maturity Onset Diabetes Mellitus':ab,ti OR 'MODY':ab,ti OR 'Diabetes Mellitus, Slow-Onset':ab,ti OR 'Diabetes Mellitus, Slow Onset':ab,ti OR 'Slow-Onset Diabetes Mellitus':ab,ti OR 'Type 2 Diabetes Mellitus':ab,ti OR 'Noninsulin-Dependent Diabetes Mellitus':ab,ti OR 'Noninsulin Dependent Diabetes Mellitus':ab,ti OR 'Maturity-Onset Diabetes':ab,ti OR 'Diabetes, Maturity-Onset':ab,ti OR 'Type 2 Diabetes':ab,ti OR 'Diabetes, Type 2':ab,ti OR 'Diabetes Mellitus, Noninsulin-Dependent':ab,ti OR 'T2DM':ab,ti |
| **6** | #4 OR #5 |
| **7** | 'sarcopenia'/exp |
| **8** | 'Sarcopenias':ab,ti OR 'muscle mass':ab,ti OR 'skeletal muscle':ab,ti OR 'skeletal muscle mass':ab,ti OR 'muscle tissue':ab,ti OR 'lean mass':ab,ti OR 'hand strength':ab,ti OR 'handgrip strength':ab,ti OR 'muscle strength':ab,ti OR 'gait speed':ab,ti OR 'muscle wasting':ab,ti OR 'muscle atrophy':ab,ti |
| **9** | #7 OR #8 |
| **10** | 'randomized controlled trial'/exp |
| **11** | 'Controlled Clinical Trial':ab,ti OR 'RCT':ab,ti OR 'randomized':ab,ti OR 'randomised':ab,ti OR 'randomly':ab,ti |
| **12** | #10 OR #11 |
| **13** | #3 AND #6 AND #9 AND #12 |

**Table S1.4** Search strategy of Cochrane Library

| **#** | **Searches** |
| --- | --- |
| **1** | MeSH descriptor: [Sodium-Glucose Transporter 2 Inhibitors] explode all trees |
| **2** | (Sodium-Glucose Transporter 2 Inhibitor):ti,ab,kw OR (Sodium Glucose Transporter 2 Inhibitors):ti,ab,kw OR (Sodium Glucose Transporter 2 Inhibitor):ti,ab,kw OR (sodium glucose cotransporter 2 inhibitor):ti,ab,kw OR (SGLT-2 Inhibitors):ti,ab,kw OR (SGLT-2 Inhibitor):ti,ab,kw OR (SGLT 2 Inhibitors):ti,ab,kw OR (SGLT 2 Inhibitor):ti,ab,kw OR (SGLT2 Inhibitors):ti,ab,kw OR (SGLT2 Inhibitor):ti,ab,kw OR (Inhibitor, SGLT-2):ti,ab,kw OR (Inhibitor, SGLT2):ti,ab,kw OR (Canagliflozin):ti,ab,kw OR (Dapagliflozin):ti,ab,kw OR (Empagliflozin):ti,ab,kw OR (Ertugliflozin):ti,ab,kw OR (Luseogliflozin):ti,ab,kw OR (Tofogliflozin):ti,ab,kw OR (Ipragliflozin):ti,ab,kw OR (Sotagliflozin):ti,ab,kw OR (Henagliflozin):ti,ab,kw OR (Remogliflozin):ti,ab,kw OR (Enavogliflozin):ti,ab,kw OR (Bexagliflozin):ti,ab,kw |
| **3** | #1 OR #2 |
| **4** | MeSH descriptor: [Diabetes Mellitus, Type 2] explode all trees |
| **5** | (Diabetes Mellitus, Stable):ti,ab,kw OR (Stable Diabetes Mellitus):ti,ab,kw OR (Diabetes Mellitus, Noninsulin Dependent):ti,ab,kw OR (Diabetes Mellitus, Adult-Onset):ti,ab,kw OR (Adult-Onset Diabetes Mellitus):ti,ab,kw OR (Diabetes Mellitus, Adult Onset):ti,ab,kw OR (Diabetes Mellitus, Ketosis-Resistant):ti,ab,kw OR (Diabetes Mellitus, Ketosis Resistant):ti,ab,kw OR (Ketosis-Resistant Diabetes Mellitus):ti,ab,kw OR (Diabetes Mellitus, Non Insulin Dependent):ti,ab,kw OR (Diabetes Mellitus, Non-Insulin-Dependent):ti,ab,kw OR (Non-Insulin-Dependent Diabetes Mellitus):ti,ab,kw OR (Diabetes Mellitus, Type II):ti,ab,kw OR (NIDDM):ti,ab,kw OR (Diabetes Mellitus, Maturity-Onset):ti,ab,kw OR (Diabetes Mellitus, Maturity Onset):ti,ab,kw OR (Maturity-Onset Diabetes Mellitus):ti,ab,kw OR (Maturity Onset Diabetes Mellitus):ti,ab,kw OR (MODY):ti,ab,kw OR (Diabetes Mellitus, Slow-Onset):ti,ab,kw OR (Diabetes Mellitus, Slow Onset):ti,ab,kw OR (Slow-Onset Diabetes Mellitus):ti,ab,kw OR (Type 2 Diabetes Mellitus):ti,ab,kw OR (Noninsulin-Dependent Diabetes Mellitus):ti,ab,kw OR (Noninsulin Dependent Diabetes Mellitus):ti,ab,kw OR (Maturity-Onset Diabetes):ti,ab,kw OR (Diabetes, Maturity-Onset):ti,ab,kw OR (Maturity Onset Diabetes):ti,ab,kw OR (Type 2 Diabetes):ti,ab,kw OR (Diabetes, Type 2):ti,ab,kw OR (Diabetes Mellitus, Noninsulin-Dependent):ti,ab,kw OR (T2DM):ti,ab,kw |
| **6** | #4 OR #5 |
| **7** | MeSH descriptor: [sarcopenia] explode all trees |
| **8** | (Sarcopenias):ti,ab,kw OR (muscle mass):ti,ab,kw OR (skeletal muscle):ti,ab,kw OR (Skeletal Muscle Mass):ti,ab,kw OR (Muscle Tissue):ti,ab,kw OR (Lean Mass):ti,ab,kw OR (Hand Strength):ti,ab,kw OR (handgrip strength):ti,ab,kw OR (muscle strength):ti,ab,kw OR (gait speed):ti,ab,kw OR (muscle wasting):ti,ab,kw OR (muscle atrophy):ti,ab,kw |
| **9** | #7 OR #8 |
| **10** | MeSH descriptor: [randomized controlled trial] explode all trees |
| **11** | (Controlled Clinical Trial):ti,ab,kw OR (RCT):ti,ab,kw OR (randomized):ti,ab,kw OR (randomised):ti,ab,kw OR (randomly):ti,ab,kw |
| **12** | #10 OR #11 |
| **13** | #3 AND #6 AND #9 AND #12 |

**Table S1.5** Search strategy of Chinese databases

钠-葡萄糖共转运蛋白2抑制剂 OR 钠-葡萄糖协同转运蛋白2抑制剂 OR SGLT2抑制剂OR 卡格列净OR达格列净OR恩格列净OR艾格列净OR艾托格列净OR鲁格列净OR托格列净OR伊格列净OR索格列净OR恒格列净OR瑞格列净OR依那格列净OR贝格列净 OR 糖尿病, 2型OR 2型糖尿病OR T2DMOR非胰岛素依赖型糖尿病OR糖尿病, 2型OR肌少症OR肌肉质量OR骨骼肌质量OR瘦体重OR瘦组织OR握力OR步速OR肌肉力量OR随机对照试验OR随机OR RCT

**Appendix 2: Characteristics of included studies**

Baseline characteristics of included studies

| Study | Design | Sample (S;C) | Age, y  (S;C) | HbA1c, % (S;C) | BMI, kg/m^2^  (S;C) | Interventions  (S;C) | Measurement  method | Duration | Background hypoglycemic intervention |
| --- | --- | --- | --- | --- | --- | --- | --- | --- | --- |
| Blonde L (2016) | RCT | S1: 483  S2: 485  C: 482 | 56.4 ± 9.5  55.8 ± 9.2  56.3 ± 9.0 | 7.8 ± 0.8  7.8 ± 0.8  7.8 ± 0.8 | 31.0 ± 5.3  31.2 ± 5.4  30.9 ± 5.5 | Canagliflozin 100mg  Canagliflozin 300mg  Glimepiride | DXA | 52 weeks | Metformin |
|  |  | S1: 63  S2: 73  C: 75 | 64.3 ± 6.6  63.0 ± 6.0  64.2 ± 6.4 | 7.8 ± 0.8  7.8 ± 0.8  7.8 ± 0.7 | 30.9 ± 4.8  31.6 ± 4.3  32.0 ± 5.5 | Canagliflozin 100mg  Canagliflozin 300mg  Placebo | DXA | 26 weeks | Conventional hypoglycemic treatment |
| Bolinder J (2014) | RCT | S: 89  C: 91 | 60.6 ± 8.2  60.8 ± 6.9 | 7.19 ± 0.44  7.16 ± 0.53 | 32.1 ± 3.9  31.7 ± 3.9 | Dapagliflozin 10mg  Placebo | DXA | 102 weeks | Metformin |
| Cefalu WT (2013) | RCT | S1: 483  S2: 485  C: 482 | 56.4 ± 9.5  55.8 ± 9.2  56.3 ± 9.0 | 7.8 ± 0.8  7.8 ± 0.8  7.8 ± 0.8 | 31.0 ± 5.3  31.2 ± 5.4  30.9 ± 5.5 | Canagliflozin 100mg  Canagliflozin 300mg  Glimepiride | DXA | 52 weeks | Metformin |
| Chehrehgosha H (2021) | RCT | S: 35  C1: 34  C2: 37 | 50.5 ± 8.4  52.5 ± 7.9  51.8 ± 7.8 | 8.08 ± 0.92  8.23 ± 0.79  7.96 ± 0.62 | 30.9 ± 3.3  29.4 ± 3.7  30.2 ± 4.4 | Empagliflozin 10mg  Pioglitazone  Placebo | DXA | 24 weeks | Conventional hypoglycemic treatment |
| Fadini GP (2017) | RCT | S: 15  C: 16 | 66.3 ± 1.8  61.0 ± 1.8 | 8.2 ± 0.2  8.2 ± 0.2 | 28.4 ± 1.4  32.8 ± 1.4 | Dapagliflozin 10mg  Placebo | BIA | 12 weeks | Conventional hypoglycemic treatment |
| Fukada H (2025) | RCT | S: 13  C:11 | 58.8 ± 16.1  57.6 ± 13.5 | 6.8 ± 0.4  6.8 ± 0.4 | 29.5 ± 3.7  30.7 ± 6.5 | Dapagliflozin 5mg  Vit E | BIA | 24 weeks | Conventional hypoglycemic treatment |
| Han E  (2020) | RCT | S: 29  C: 15 | 52.5 ± 10.3  56.7 ± 11.8 | 6.7 ± 0.7  6.6 ± 0.6 | 30.4 ± 5.4  30.2 ± 2.5 | Ipragliflozin 50mg  Placebo | DXA | 24 weeks | Conventional hypoglycemic treatment |
| Hiruma S (2023) | RCT | S: 23  C: 19 | 52.4 ± 10.2  50.0 ± 11.9 | 7.1 ± 0.8  7.2 ± 1.0 | 30.6 ± 5.0  28.6 ± 5.2 | Empagliflozin 10mg  Sitagliptin | DXA | 12 weeks | α-glucosidase inhibitors, sulphonylureas, glinides |
| Horibe K (2022) | RCT | S: 26  C: 24 | 59.7±12.0  62.3±6.5 | 7.68± 0.53  7.73 ± 0.50 | 28.0 ± 4.0  27.6 ± 3.8 | Dapagliflozin 5mg  Placebo | BIA | 24 weeks | Conventional hypoglycemic treatment |
| Hoshika Y (2022) | RCT | S: 25  C: 30 | 67.5 ± 8.8  67.0 ± 10.9 | 6.7±0.8  6.8±1.0 | 24.5 ± 3.3  25.2 ± 4.4 | Empagliflozin 10mg  Placebo | BIA | 24 weeks | Conventional hypoglycemic treatment |
| Igarashi H (2023) | RCT | S: 8  C: 8 | 53.8 ± 15.2  50.4 ± 14.0 | 11.8±1.3  12.9±1.8 | 26.3 ± 5.1  27.7 ± 5.1 | Canagliflozin 100mg  Placebo | BIA | 12 weeks | Insulin |
| Inoue H  (2019) | RCT | S: 24  C: 24 | 60.5 ±9.8  60.8 ±12.1 | 8.12±0.93  8.30±0.65 | 27.9± 4.0  27.7±4.5 | Ipragliflozin 50mg  Placebo | BIA | 24 weeks | Conventional hypoglycemic treatment |
| Kang SM (2023) | RCT | S: 70  C: 70 | 55.7 ± 9.9  54.2 ± 8.9 | 8.5±0.8  8.5±0.7 | 26.8 ± 3.6  26.5 ± 3.6 | Ipragliflozin 50mg  Sitagliptin | BIA | 24 weeks | Metformin and sulphonylurea |
| Kayano H (2020) | RCT | S: 36  C: 28 | 69.4 ± 7.1  66.0 ± 9.5 | 7.6±0.7  7.5±0.6 | 25.6 ± 4.2  25.8 ± 3.9 | Dapagliflozin 5mg  Placebo | BIA | 24 weeks | Conventional hypoglycemic treatment |
| Kitazawa T (2020) | RCT | S: 33  C: 31 | 57.3 ± 11.4  57.6 ± 9.3 | 7.4±0.5  7.5±0.4 | 25.3±3.9  25.4±3.8 | Tofogliflozin 20mg  Glimepiride | BIA | 24 weeks | Metformin and DPP-4i |
| Koshizaka M (2019) | RCT | S: 48  C: 50 | 56.6 ± 11.9  55.7 ± 12.2 | 7.95 ± 0.73  8.12 ± 0.90 | 27.55 ± 4.24  28.83 ± 5.32 | Ipragliflozin 50mg  Metformin | CT | 24 weeks | Sitagliptin |
| Koshizaka M (2021) | RCT | S: 15  C: 14 | 68.7±2.4  69.4±2.9 | 7.9±0.7  8.0±0.9 | 26.8±3.8  27.1±3.9 | Ipragliflozin 50mg  Metformin | CT | 24 weeks | Sitagliptin |
| Koshizaka M (2021) | RCT | S: 48  C: 50 | 56.6 ± 11.9  55.7 ± 12.2 | 7.95 ± 0.73  8.12 ± 0.90 | 27.55 ±4.24  28.83±5.32 | Ipragliflozin 50mg  Metformin | CT | 24 weeks | Sitagliptin |
| McCrimmon RJ (2020) | RCT | S: 90  C: 88 | 58.6±10.1  57.8±9.9 | 8.3 ±1.0  8.5 ± 1.1 | 32.3±5.5  32.6±6.4 | Canagliflozin 300mg  Semaglutide | DXA | 52 weeks | Metformin |
| Nakaguchi H (2020) | RCT | S: 31  C: 30 | 66.3±9.5  67.2±9.0 | 8.08±0.76  8.04±0.75 | 25.8±4.1  26.4±4.6 | Empagliflozin 10mg  Liraglutide | DXA | 24 weeks | Conventional hypoglycemic treatment |
| Park HK (2023) | RCT | S: 56  C: 56 | 54.8 ± 9.0  55.5 ± 9.3 | 8.0 ± 0.7  8.0 ± 0.7 | 27.0 ± 3.2  26.7 ± 3.6 | Dapagliflozin 10mg  Glimepiride | DXA | 52 weeks | Metformin |
| Sargeant JA (2022) | RCT | S1: 17  C1: 17 | 61.0 ± 8.15  63.0 ± 9.63 | 6.9 ± 0.44  7.0 ± 0.59 | 31.4 ± 3.93  32.8 ± 4.59 | Empagliflozin 25mg  Placebo | DXA | 24 weeks | Metformin andusual diet |
|  |  | S2: 17  C2: 17 | 65.0 ± 0.37  63.0 ± 6.67 | 6.8 ± 0.37  6.9 ± 0.67 | 31.2 ± 4.07  31.9 ± 6.22 | Empagliflozin 25mg  Placebo | DXA | 24 weeks | Metformin and energy-restricted diet |
| Shimizu M (2019) | RCT | S: 33  C: 24 | 56.2 ± 11.5  57.1± 13.8 | 7.96 ± 1.40  7.70 ± 0.55 | 27.6 ± 4.7  28.3 ± 3.5 | Dapagliflozin 5mg  Placebo | BIA | 24 weeks | Conventional hypoglycemic treatment |
| Tsukagoshi-Yamaguchi A (2023) | RCT | S: 15  C: 15 | 57.8 ± 13.3  59.1 ± 9.6 | 8.1 ± 0.7  8.1 ± 1.0 | 29.0 ± 5.7  28.1 ± 5.1 | Ipragliflozin 50mg  Metformin | CT | 24 weeks | Sitagliptin |
| Tsurutani Y (2018) | RCT | S: 60 C: 59 | 53.5 ± 11.72  54.0 ± 10.7 | 7.6±1.19  7.4±0.89 | 28.8±4.67  28.5±3.85 | Ipragliflozin 50mg  Sitagliptin | BIA | 12 weeks | Conventional hypoglycemic treatment |
| Wolf VLW (2021) | RCT | S: 44 C: 45 | 58.0 ± 7.0  58.0 ± 7.0 | 7.7±1.2  7.9±1.4 | 30.0±7.0  31.0±7.0 | Dapagliflozin 10mg  Glibenclamide | DXA | 12 weeks | Metformin |
| Yabe D (2023) | RCT | S: 64 C: 63 | 74.2 ± 4.9  74.0 ± 5.1 | 7.6±0.5  7.6±0.5 | 25.7±3.3  25.4±2.6 | Empagliflozin 10mg  Placebo | BIA | 52 weeks | Conventional hypoglycemic treatment |
| Yamakage H (2020) | RCT | S: 27 C: 27 | 58.4±13.0  60.7±11.9 | 7.5±0.8  7.4±0.9 | 31.3±7.6  30.7±6.2 | Dapagliflozin 5mg  Placebo | BIA | 24 weeks | Conventional hypoglycemic treatment |
| Zeng YH (2022) | RCT | S: 46 C: 51 | 58.9 ± 9.9  58.7±10.2 | 9.2±1.4  9.0±1.2 | 27.7±5.0  28.0±3.5 | Empagliflozin 25mg  Linagliptin | BIA | 24 weeks | Insulin |
| Jiang LJ (2019) | RCT | S: 30 C: 30 | 49.63± 6.36  50.80 ± 2.10 | 8.91 ± 1.43  8.54 ± 1.28 | NR | Dapagliflozin 10mg  Placebo | DXA | 12 weeks | Conventional hypoglycemic treatment |
| Liang Y | RCT | S: 30 C: 30 | 49.7 ± 12.4  51.5 ± 10.8 | 8.2 ± 0.9  8.1 ± 1.3 | 29.4 ± 4.3  28.5 ± 4.2 | Dapagliflozin 10mg  Insulin | DXA | 12 weeks | Metformin |
| Zhang BQ | RCT | S: 20 C: 20 | 59.3 ± 8.5  56.0 ± 9.3 | 8.8 ± 1.6  8.7 ± 1.7 | 22.8 ± 1.6  22.1 ± 1.0 | Canagliflozin 100mg  Loxenatide | DXA | 16 weeks | Metformin |
| Zhang JW | RCT | S: 31 C: 16 | 46.87±10.63  54.75±7.32 | 7.91±1.46  7.26±1.23 | 28.72±4.37  27.56±3.86 | Dapagliflozin 10mg  Placebo | BIA | 24 weeks | Metformin |

Abbreviations: DXA, dual-energy X- ray absorptiometry; BIA, bioelectrical impedance analysis; CT, computed tomography; NR, not reported

**Appendix 3: Risk of bias of randomized clinical trials**

Figure S3: Overall risk of bias presented as percentage of each risk of bias item across all included studies.

Green = Low risk, Red = High risk, Yellow = Some concerns.

As percentage

|  | Low risk | Some concerns | High risk |
| --- | --- | --- | --- |
| Overall Bias | 27.27 | 69.7 | 3.03 |
| Randomization process | 96.97 | 0 | 3.03 |
| Deviations from intended interventions | 27.27 | 69.7 | 3.03 |
| Missing outcome data | 93.34 | 6.06 | 0 |
| Measurement of the outcome | 96.97 | 3.03 | 0 |
| Selection of the reported result | 87.88 | 12.12 | 0 |

Table S3: Study level risk of bias assessment using Cochrane risk of bias tool 2.0 for assessing risk of bias of randomized clinical trials.

| Unique ID | Study ID | Randomizationprocess | Deviationsfromintendedinterventions | Missing outcomedata | Measurement ofthe outcome | Selection of thereported result | Over all |
| --- | --- | --- | --- | --- | --- | --- | --- |
| Blonde L (2016) | doi:10.1080/00325481.2016.1169894 | Low | Low | Low | Low | Low | Low |
| Bolinder J (2014) | doi:10.1111/dom.12189 | Low | Low | Low | Low | Low | Low |
| Cefalu WT (2013) | doi:10.1016/S0140-6736(13)60683-2 | Low | Low | Low | Low | Low | Low |
| Chehrehgosha H (2021) | doi:10.1007/s13300-021-01011-3 | Low | Low | Low | Low | Low | Low |
| Fadini GP (2017) | doi:10.1186/s12933-017-0529-3 | Low | Low | Low | Low | Low | Low |
| Fukada H (2025) | doi:10.3389/fmed.2025.1542741 | Low | Some concerns | Low | Low | Low | Some concerns |
| Han E (2020) | doi:10.3390/jcm9010259 | Low | Some concerns | Low | Low | Low | Some concerns |
| Hiruma S (2023) | doi:10.1111/dom.15006 | Low | Some concerns | Low | Low | Low | Some concerns |
| Horibe K (2022) | doi:10.1016/j.diabres.2022.109781 | Low | Some concerns | Low | Low | Low | Some concerns |
| Hoshika Y (2022) | doi:10.1016/j.cardfail.2021.07.022 | Low | Low | Low | Low | Low | Low |
| Igarashi H (2023) | doi:10.1186/s13098-023-00990-6 | Low | Some concerns | Low | Low | Low | Some concerns |
| Inoue H (2019) | doi:10.1111/jdi.12985 | Low | Some concerns | Low | Low | Low | Some concerns |
| Kang SM (2023) | doi:10.1111/dom.15056 | Low | Some concerns | Low | Low | Low | Some concerns |
| Kayano H (2020) | doi:10.1253/circj.CJ-20-0341 | Low | Some concerns | Low | Low | Low | Some concerns |
| Kitazawa T (2020) | doi:10.1111/dom.14059 | Low | Some concerns | Low | Low | Low | Some concerns |
| Koshizaka M (2019) | doi:10.1111/dom.13750 | Low | Some concerns | Low | Low | Low | Some concerns |
| Koshizaka M (2021) | doi:10.1007/s13300-020-00949-0 | Low | Some concerns | Low | Low | Low | Some concerns |
| Koshizaka M (2021) | doi:10.1111/jdi.13340 | Low | Some concerns | Low | Low | Some concerns | Some concerns |
| McCrimmon RJ (2020) | doi:10.1007/s00125-019-05065-8 | Low | Low | Low | Low | Low | Low |
| Nakaguchi H (2020) | doi:10.1111/jdi.13270 | Low | Some concerns | Low | Low | Low | Some concerns |
| Park HK (2023) | doi:10.1111/dom.15164 | Low | Some concerns | Low | Low | Low | Some concerns |
| Sargeant JA (2022) | doi:10.1111/dom.14721 | Low | Low | Low | Low | Low | Low |
| Shimizu M (2019) | doi:10.1111/dom.13520 | Low | Some concerns | Low | Low | Low | Some concerns |
| Tsukagoshi-Yamaguchi A (2023) | doi:10.1002/phar.2884 | Low | Some concerns | Low | Low | Low | Some concerns |
| Tsurutani Y (2018) | doi:10.1111/dom.13421 | Low | Some concerns | Low | Low | Low | Some concerns |
| Wolf VLW (2021) | doi:10.1038/s41387-021-00160-5 | Low | Some concerns | Low | Low | Low | Some concerns |
| Yabe D (2023) | doi:10.1111/dom.15249 | Low | Low | Low | Low | Low | Low |
| Yamakage H (2020) | doi:10.1111/jdi.13179 | Low | Some concerns | Low | Low | Low | Some concerns |
| Zeng YH (2022) | doi:10.1038/s41598-022-21486-9 | Low | Some concerns | Low | Low | Low | Some concerns |
| Jiang LJ (2019) | NA | Low | Some concerns | Low | Low | Some concerns | Some concerns |
| Liang Y(2021) | NA | Low | Some concerns | Low | Low | Some concerns | Some concerns |
| Zhang BQ (2024) | NA | Low | Some concerns | Some concerns | Low | Low | Some concerns |
| Zhang JW (2023) | NA | High | High | Some concerns | Some concerns | Some concerns | High |

**Appendix 4: Evaluation of inconsistency and heterogeneity**

Table S4.1: Global consistency

| Clinical outcomes | Chi square | P value | τ^2^ |
| --- | --- | --- | --- |
| **Primary outcomes** |  | | |
| SMM | 0.05 | 0.8299 | 0.03 |
| LM | 0.09 | 0.7620 | 0.05 |
| **Secondary outcomes** |  | | |
| BW | 1.42 | 0.4922 | 0.02 |
| BMI | 0.96 | 0.3263 | 0.02 |
| WC | 250.12 | 0.0723 | 0.03 |
| FM | 11.12 | 0.0835 | 0.24 |
| PBF | 11.75 | 0.0612 | 0.03 |
| VFA | 249.48 | 0.0712 | 0.03 |
| SFA | 18.44 | 0.0625 | 0.03 |
| TBW | 1.08 | 0.2995 | 0.44 |
| **Safety outcomes** |  | | |
| Any AE | 2.13 | 0.5452 | 0.03 |
| Any serious AE | 0.19 | 0.6637 | 0.02 |
| AE leading to discontinuation | 1.52 | 0.2182 | 0.03 |
| Genitourinary tract infection | 2.76 | 0.0967 | 0.03 |

Abbreviations:SMM, skeletal muscle mass; LM, lean mass; BW, body weight; BMI, body mass index; WC, waist circumference; FM, fat mass; PBF, percentage body fat; VFA, visceral fat area; SFA, subcutaneous fat area; TBW, total body water; AE, adverse events.

Table S4.2: Side-splitting of Lean mass. Inconsistency test between direct and indirect treatment comparisons in mixed treatment comparison.

| Comparison | Direct | | Indirect | | Difference | | |
| --- | --- | --- | --- | --- | --- | --- | --- |
|  | Coef. | Std.Err. | Coef. | Std.Err. | Coef. | Std.Err. | P>\|z\| |
| Canagliflozin v.s. Glimepiride | 2.099916 | 0.3561773 | 1.860549 | 0.7046744 | 0.2393674 | 0.789575 | 0.762 |
| Canagliflozin v.s. Placebo | 0.7502056 | 0.3560674 | 0.9892761 | 0.7045458 | -0.2390705 | 0.7894109 | 0.762 |
| Dapagliflozin v.s. Glibenclamide | 1.276 | 0.3205891 | -0.0561836 | 200.1261 | 1.332183 | 200.1261 | 0.995 |
| Dapagliflozin v.s. Glimepiride | 1.280007 | 0.521453 | 1.519237 | 0.5929152 | -0.2392297 | 0.7895965 | 0.762 |
| Dapagliflozin v.s. Insulin | 0.203 | 1.229151 | -1.129196 | 200.0147 | 1.332196 | 200.0111 | 0.995 |
| Dapagliflozin v.s. Placebo | 0.169533 | 0.3129381 | -0.0695212 | 0.7249152 | 0.2390542 | 0.7895794 | 0.762 |
| Empagliflozin v.s. Liraglutide | 0.1 | 0.5442653 | 0.9368205 | 200.0758 | -0.8368205 | 200.0753 | 0.997 |
| Empagliflozin v.s. Placebo | 1.217263 | 0.2535357 | 0.5895965 | 70.72404 | 0.6276665 | 70.72487 | 0.993 |
| Glimepiride v.s. Tofogliflozin | -2.2 | 0.4861292 | -6.302501 | 199.8766 | 4.102501 | 199.8762 | 0.984 |
| Ipragliflozin v.s. Sitagliptin | 0.3 | 1.725345 | 0.6885211 | 199.9707 | -0.3885211 | 199.9634 | 0.998 |
| Ipragliflozin v.s. Placebo | 0.9930959 | 0.2329308 | 0.7342237 | 81.64657 | 0.2588721 | 81.64707 | 0.997 |

Table S4.3: Side-splitting of Fat mass. Inconsistency test between direct and indirect treatment comparisons in mixed treatment comparison.

| Comparison | Direct | | Indirect | | Difference | | |
| --- | --- | --- | --- | --- | --- | --- | --- |
|  | Coef. | Std.Err. | Coef. | Std.Err. | Coef. | Std.Err. | P>\|z\| |
| Canagliflozin v.s. Glimepiride | 1.035965 | 0.1376473 | 3.085352 | 0.6148082 | -2.049387 | 0.6300285 | 0.052 |
| Canagliflozin v.s. Placebo | 1.824039 | 0.2992727 | -0.2248664 | 0.5543654 | 2.048906 | 0.6299882 | 0.061 |
| Dapagliflozin v.s. Glibenclamide | 2.372 | 0.5623277 | 3.197641 | 200.1591 | -0.8256412 | 200.1595 | 0.997 |
| Dapagliflozin v.s. Glimepiride | 2.589996 | 0.3817434 | 0.5404771 | 0.5012243 | 2.049519 | 0.6300439 | 0.067 |
| Dapagliflozin v.s. Insulin | 6.118 | 1.542816 | 6.94361 | 200.011 | -0.8256102 | 200.0063 | 0.997 |
| Dapagliflozin v.s. Placebo | 1.328428 | 0.377835 | 3.377958 | 0.5042049 | -2.04953 | 0.6300671 | 0.056 |
| Empagliflozin v.s. Linagliptin | 0.66 | 0.7760684 | 0.522072 | 199.995 | 0.137928 | 199.9947 | 0.999 |
| Empagliflozin v.s. Liraglutide | 0.1000001 | 0.5667288 | -0.0379431 | 200.1068 | 0.1379432 | 200.1072 | 0.999 |
| Empagliflozin v.s. Pioglitazone | 1.800015 | 0.9278725 | 2.970536 | 1.992303 | -1.170521 | 2.165683 | 0.589 |
| Empagliflozin v.s. Placebo | 1.268466 | 0.4030222 | 1.363987 | 55.47368 | -0.0955207 | 55.47591 | 0.999 |
| Glimepiride v.s. Tofogliflozin | -1.4 | 0.6503749 | -4.075615 | 199.8416 | 2.675615 | 199.8418 | 0.989 |
| Ipragliflozin v.s. Sitagliptin | 1.2 | 1.218051 | 1.956221 | 199.9969 | -0.7562208 | 199.9944 | 0.997 |
| Ipragliflozin v.s. Placebo | 1.715562 | 0.5839081 | 1.211381 | 81.65498 | 0.504181 | 81.65797 | 0.995 |
| Pioglitazone v.s. Placebo | -1.02 | 1.01991 | 0.1506126 | 1.852463 | -1.170613 | 2.165734 | 0.589 |

Table S4.4: Side-splitting of VFA. Inconsistency test between direct and indirect treatment comparisons in mixed treatment comparison.

| Comparison | Direct | | Indirect | | Difference | | |
| --- | --- | --- | --- | --- | --- | --- | --- |
|  | Coef. | Std.Err. | Coef. | Std.Err. | Coef. | Std.Err. | P>\|z\| |
| Dapagliflozin v.s. Placebo | 11.70139 | 4.58873 | 30.17168 | 37.87835 | -18.47029 | 38.15529 | 0.628 |
| Empagliflozin v.s. Placebo | 12.2 | 12.13944 | 11.73611 | 200.5753 | 0.4638889 | 201.6743 | 0.998 |
| Ipragliflozin v.s. Metformin | 22.31728 | 5.77162 | 64.85071 | 116.0494 | -42.53342 | 115.9058 | 0.714 |
| Ipragliflozin v.s. Sitagliptin | 8.5 | 6.55992 | 50.81999 | 200.3565 | -42.31999 | 200.2491 | 0.833 |
| Ipragliflozin v.s. Placebo | 33.12706 | 2.096594 | 9.344131 | 47.41516 | 23.78293 | 47.47169 | 0.616 |
| Pioglitazone v.s. Placebo | -2.1 | 12.16071 | -2.563889 | 201.1266 | 0.463889 | 201.6748 | 0.998 |

Table S4.5: Side-splitting of BW. Inconsistency test between direct and indirect treatment comparisons in mixed treatment comparison.

| Comparison | Direct | | Indirect | | Difference | | |
| --- | --- | --- | --- | --- | --- | --- | --- |
|  | Coef. | Std.Err. | Coef. | Std.Err. | Coef. | Std.Err. | P>\|z\| |
| Canagliflozin v.s. Glimepiride | 4.595048 | 0.1917103 | 3.951875 | 0.7334818 | 0.6431724 | 0.7581216 | 0.396 |
| Canagliflozin v.s. Placebo | 2.639678 | 0.3993349 | 3.281619 | 0.6443031 | -0.6419411 | 0.7580204 | 0.397 |
| Dapagliflozin v.s. Glibenclamide | 3.801 | 0.5478711 | 2.547013 | 200.0869 | 1.253987 | 200.0862 | 0.995 |
| Dapagliflozin v.s. Glimepiride | 3.640008 | 0.5064743 | 4.282942 | 0.5641505 | -0.6429337 | 0.7581459 | 0.396 |
| Dapagliflozin v.s. Placebo | 2.327441 | 0.3494819 | 1.684635 | 0.672821 | 0.6428052 | 0.7581763 | 0.397 |
| Empagliflozin v.s. Linagliptin | 1.85 | 0.3827336 | 0.6796661 | 199.7517 | 1.170334 | 199.7514 | 0.995 |
| Empagliflozin v.s. Liraglutide | 0.2 | 0.4998345 | -0.9704679 | 200.3855 | 1.170468 | 200.3849 | 0.995 |
| Empagliflozin v.s. Sitagliptin | 1.600024 | 0.7440329 | 2.333162 | 0.4644684 | -0.7331379 | 0.8771061 | 0.403 |
| Empagliflozin v.s. Placebo | 2.322312 | 0.3067106 | 1.589496 | 0.8216355 | 0.7328157 | 0.877021 | 0.403 |
| Glimepiride v.s. Tofogliflozin | -3.6 | 0.4225972 | -12.70798 | 199.8636 | 9.107983 | 199.8632 | 0.964 |
| Ipragliflozin v.s. Metformin | 1.626583 | 0.3889781 | 0.0502898 | 141.3392 | 1.576294 | 141.3387 | 0.991 |
| Ipragliflozin v.s. Sitagliptin | 2.012467 | 0.3036427 | 1.279263 | 0.8228636 | 0.7332044 | 0.8770962 | 0.403 |
| Ipragliflozin v.s. Placebo | 2.00161 | 0.171652 | 2.73485 | 0.8600074 | -0.7332393 | 0.8769717 | 0.403 |

Table S4.6: Side-splitting of BMI. Inconsistency test between direct and indirect treatment comparisons in mixed treatment comparison.

| Comparison | Direct | | Indirect | | Difference | | |
| --- | --- | --- | --- | --- | --- | --- | --- |
|  | Coef. | Std.Err. | Coef. | Std.Err. | Coef. | Std.Err. | P>\|z\| |
| Canagliflozin v.s. Glimepiride | 1.700052 | 0.1000899 | -0.6297149 | 2.37243 | 2.329767 | 2.37454 | 0.327 |
| Canagliflozin v.s. Placebo | -1.1 | 2.36766 | 1.245682 | 0.2815012 | -2.345682 | 2.384335 | 0.325 |
| Dapagliflozin v.s. Glibenclamide | 1.4 | 0.1937376 | 0.7776791 | 200.9569 | 0.6223209 | 200.9568 | 0.998 |
| Dapagliflozin v.s. Glimepiride | 1.369999 | 0.1889916 | 3.701 | 2.368492 | -2.331002 | 2.376023 | 0.327 |
| Dapagliflozin v.s. Insulin | 4.7 | 1.129798 | 4.077666 | 199.9495 | 0.6223335 | 199.9463 | 0.998 |
| Dapagliflozin v.s. Placebo | 0.9156527 | 0.1830634 | -1.422282 | 2.372457 | 2.337934 | 2.3795 | 0.326 |
| Empagliflozin v.s. Liraglutide | 0.1 | 0.1421248 | -0.3261334 | 198.964 | 0.4261334 | 198.9639 | 0.998 |
| Empagliflozin v.s. Sitagliptin | 0.6000003 | 0.2654384 | 0.8508027 | 89.29809 | -0.2508024 | 89.29864 | 0.998 |
| Glimepiride v.s. Tofogliflozin | -1.3 | 0.1538304 | -4.691196 | 200.0843 | 3.391196 | 200.0842 | 0.986 |
| Ipragliflozin v.s. Metformin | 0.634375 | 0.1499901 | -0.1916109 | 141.0882 | 0.8259859 | 141.0881 | 0.995 |
| Ipragliflozin v.s. Sitagliptin | 0.400004 | 0.5407641 | 0.1183396 | 66.76805 | 0.2816644 | 66.76975 | 0.997 |
| Ipragliflozin v.s. Placebo | 0.8000001 | 0.0548778 | 1.118172 | 46.4079 | -0.3181722 | 46.40794 | 0.995 |

Table S4.7: Side-splitting of Any AE. Inconsistency test between direct and indirect treatment comparisons in mixed treatment comparison.

| Comparison | Direct | | Indirect | | Difference | | |
| --- | --- | --- | --- | --- | --- | --- | --- |
|  | Coef. | Std. Err. | Coef. | Std. Err. | Coef. | Std. Err. | P>\|z\| |
| Canagliflozin v.s. Glimepiride | 0.1222851 | 0.0761694 | 0.1084524 | 0.4973609 | 0.0138328 | 0.5030829 | 0.978 |
| Canagliflozin v.s. Placebo | -0.2162626 | 0.1982227 | -0.2023639 | 0.4623631 | -0.0138988 | 0.5030372 | 0.978 |
| Dapagliflozin v.s. Glimepiride | -0.0289866 | 0.3703046 | -0.0151583 | 0.3405495 | -0.0138283 | 0.5030907 | 0.978 |
| Dapagliflozin v.s. Placebo | -0.3536979 | 0.2664425 | -0.3675136 | 0.4268684 | 0.0138157 | 0.5030962 | 0.978 |
| Empagliflozin v.s. Pioglitazone | -0.2876774 | 0.8050622 | 0.4225241 | 1.568006 | -0.7102014 | 1.714656 | 0.679 |
| Empagliflozin v.s. Sitagliptin | -1.988905 | 0.8541319 | -0.2810175 | 0.9185751 | -1.707888 | 1.254311 | 0.173 |
| Empagliflozin v.s. Placebo | 0.021895 | 0.2882518 | -1.68565 | 1.22058 | 1.707545 | 1.254165 | 0.173 |
| Glimepiride v.s. Tofogliflozin | -0.071459 | 0.7559893 | -0.3136425 | 200.0101 | 0.2421835 | 200.0086 | 0.999 |
| Ipragliflozin v.s. Metformin | -0.6636799 | 0.6851328 | 0.5687735 | 141.4417 | -1.232453 | 141.44 | 0.993 |
| Ipragliflozin v.s. Sitagliptin | -0.6393099 | 0.282814 | -2.34724 | 1.222069 | 1.70793 | 1.254367 | 0.173 |
| Ipragliflozin v.s. Placebo | -0.3364971 | 0.8251914 | 1.3709 | 0.9446093 | -1.707397 | 1.254296 | 0.173 |
| Pioglitazone v.s. Placebo | -0.0923733 | 0.853591 | 0.6178457 | 1.48905 | -0.710219 | 1.714699 | 0.679 |

Table S4.8: Side-splitting of AE leading to discontinuation. Inconsistency test between direct and indirect treatment comparisons in mixed treatment comparison.

| Comparison | Direct | | Indirect | | Difference | | |
| --- | --- | --- | --- | --- | --- | --- | --- |
|  | Coef. | Std.Err. | Coef. | Std.Err. | Coef. | Std.Err. | P>\|z\| |
| Canagliflozin v.s. Glimepiride | -0.0512588 | 0.1361286 | 1.268042 | 1.06328 | -1.319301 | 1.0715 | 0.218 |
| Canagliflozin v.s. Placeobo | -0.089325 | 0.2663178 | -1.408421 | 1.038206 | 1.319096 | 1.071359 | 0.218 |
| Dapagliflozin v.s. Glimepiride | -0.0173698 | 0.8411882 | -1.336735 | 0.6637143 | 1.319365 | 1.071508 | 0.218 |
| Dapagliflozin v.s. Placeobo | -1.374855 | 0.5933505 | -0.0554383 | 0.8921984 | -1.319417 | 1.071492 | 0.218 |
| Empagliflozin v.s. Sitagliptin | 0.0889475 | 2.023984 | 0.4606219 | 200.0288 | -0.3716744 | 200.0186 | 0.999 |
| Empagliflozin v.s. Placeobo | 0.0162579 | 0.8406093 | -0.2067608 | 89.43359 | 0.2230187 | 89.4391 | 0.998 |

**Appendix 5: Network plot and forest plots of secondary outcomes**

Figure S5.1: Network plot of interventions for FM, and forest plot of network effect sizes compared with placebo. The size of each node is proportional to the number of participants included in the trials, and the thickness of the connecting lines reflects the number of studies for each comparison.

Favours placebo

FavoursSGLT2 inhibitors

Negative values indicate agreater reduction in FM,favouring SGLT2 inhibitors.

Figure S5.2: Network plot of interventions for PBF, and forest plot of network effect sizes compared with placebo. The size of each node is proportional to the number of participants included in the trials, and the thickness of the connecting lines reflects the number of studies for each comparison.

FavoursSGLT2 inhibitors

Favours placebo

Negative values indicate a reduction in PBF,favouring SGLT2 inhibitors.

Figure S5.3: Network plot of interventions for VFA, and forest plot of network effect sizes compared with placebo. The size of each node is proportional to the number of participants included in the trials, and the thickness of the connecting lines reflects the number of studies for each comparison.

Favours placebo

FavoursSGLT2 inhibitors

Negative values indicate a reduction in VFA,favouring SGLT2 inhibitors.

Figure S5.4: Network plot of interventions for SFA, and forest plot of network effect sizes compared with placebo. The size of each node is proportional to the number of participants included in the trials, and the thickness of the connecting lines reflects the number of studies for each comparison.

Favours placebo

FavoursSGLT2 inhibitors

Negative values indicate a reduction in SFA,favouring SGLT2 inhibitors.

Figure S5.5: Network plot of interventions for BW, and forest plot of network effect sizes compared with placebo. The size of each node is proportional to the number of participants included in the trials, and the thickness of the connecting lines reflects the number of studies for each comparison.

FavoursSGLT2 inhibitors

Favours placebo

Negative values indicate a reduction in BW, favouring SGLT2 inhibitors.

Figure S5.6: Network plot of interventions for BMI, and forest plot of network effect sizes compared with placebo. The size of each node is proportional to the number of participants included in the trials, and the thickness of the connecting lines reflects the number of studies for each comparison.

Favours placebo

FavoursSGLT2 inhibitors

Favours placebo

FavoursSGLT2 inhibitors

Negative values indicate agreater reduction in BMI, favouring SGLT2 inhibitors.

Figure S5.7: Network plot of interventions for WC, and forest plot of network effect sizes compared with placebo. The size of each node is proportional to the number of participants included in the trials, and the thickness of the connecting lines reflects the number of studies for each comparison.

Favours placebo

FavoursSGLT2 inhibitors

Negative values indicate agreater reduction in WC, favouring SGLT2 inhibitors.

Figure S5.8: Network plot of interventions for TBW, and forest plot of network effect sizes compared with placebo. The size of each node is proportional to the number of participants included in the trials, and the thickness of the connecting lines reflects the number of studies for each comparison.

increase in TBW

decrease in TBW

Negative values indicate a reduction in TBW.

Figure S5.9: Network plot of interventions for any AE, and forest plot of network effect sizes compared with placebo. The size of each node is proportional to the number of participants included in the trials, and the thickness of the connecting lines reflects the number of studies for each comparison.

FavoursSGLT2 inhibitors

Favours placebo

Values <1 indicate lower risk of any AE; values >1 indicate higher risk.

Figure S5.10: Network plot of interventions for Any serious AE, and forest plot of network effect sizes compared with placebo. The size of each node is proportional to the number of participants included in the trials, and the thickness of the connecting lines reflects the number of studies for each comparison.

Favours placebo

FavoursSGLT2 inhibitors

Values <1 indicate lower risk of any serious AE; values >1 indicate higher risk.

Figure S5.11: Network plot of interventions for AE leading to discontinuation, and forest plot of network effect sizes compared with placebo. The size of each node is proportional to the number of participants included in the trials, and the thickness of the connecting lines reflects the number of studies for each comparison.

FavoursSGLT2 inhibitors

Favours placebo

Values <1 indicate lower risk of AE leading to discontinuation; values >1 indicate higher risk.

Figure S5.12: Network plot of interventions for genitourinary tract infection, and forest plot of network effect sizes compared with placebo. The size of each node is proportional to the number of participants included in the trials, and the thickness of the connecting lines reflects the number of studies for each comparison.

Favours placebo

FavoursSGLT2 inhibitors

Values <1 indicate lower risk of genitourinary tract infection; values >1 indicate higher risk.

**Appendix 6: SUCRA and cumulative probability plots**

Figure S6.1: Cumulative ranking curve plots of SGLT2 inhibitors for SMM in range network. Higher surface under the curve reflects higher probability of association with SMM.

Table S6.1: SUCRA of the effects of various SGLT2 inhibitors on SMM.

| Treatment | SUCRA | PrBest | MeanRank |
| --- | --- | --- | --- |
| Canagliflozin | 40.9 | 15.1 | 4.5 |
| Dapagliflozin | 46.6 | 1.5 | 4.2 |
| Empagliflozin | 33.6 | 0.1 | 5 |
| Ipragliflozin | 48.4 | 23.5 | 4.1 |
| Linagliptin | 73.6 | 30 | 2.6 |
| Loxenatide | 41.4 | 21.1 | 4.5 |
| Placebo | 65.5 | 8.6 | 3.1 |

Abbreviations: SUCRA, surface under the cumulative ranking curve.

Figure S6.2: Cumulative ranking curve plots of SGLT2 inhibitors for LM in range network. Higher surface under the curve reflects higher probability of association with LM.

Table S6.2: SUCRA of the effects of various SGLT2 inhibitors on LM.

| Treatment | SUCRA | PrBest | MeanRank |
| --- | --- | --- | --- |
| Canagliflozin | 41.5 | 0 | 7.4 |
| Dapagliflozin | 64.9 | 0 | 4.9 |
| Empagliflozin | 22.3 | 0 | 9.5 |
| Glibenclamide | 91.5 | 31.8 | 1.9 |
| Glimepiride | 93.5 | 44.9 | 1.7 |
| Insulin | 63.2 | 13 | 5 |
| Ipragliflozin | 32.6 | 0 | 8.4 |
| Liraglutide | 28.4 | 0 | 8.9 |
| Semaglutide | 12.7 | 0 | 10.6 |
| Sitagliptin | 44.8 | 10.2 | 7.1 |
| Tofogliflozin | 34.3 | 0 | 8.2 |
| Placebo | 70.4 | 0 | 4.3 |

Figure S6.3: Cumulative ranking curve plots of SGLT2 inhibitors for FM in range network. Higher surface under the curve reflects higher probability of association with FM.

Table S6.3: SUCRA of the effects of various SGLT2 inhibitors on FM.

| Treatment | SUCRA | PrBest | MeanRank |
| --- | --- | --- | --- |
| Canagliflozin | 67.9 | 0.4 | 5.5 |
| Dapagliflozin | 81 | 10.4 | 3.7 |
| Empagliflozin | 65.9 | 0.9 | 5.8 |
| Glibenclamide | 18.5 | 0 | 12.4 |
| Glimepiride | 29.8 | 0 | 10.8 |
| Insulin | 0.4 | 0 | 14.9 |
| Ipragliflozin | 79.2 | 15.3 | 3.9 |
| Linagliptin | 46.8 | 2.1 | 8.5 |
| Liraglutide | 63 | 4.7 | 6.2 |
| Loxenatide | 49.1 | 6.5 | 8.1 |
| Pioglitazone | 17.9 | 0.1 | 12.5 |
| Semaglutide | 86 | 42.7 | 3 |
| Sitagliptin | 46.6 | 7.4 | 8.5 |
| Tofogliflozin | 69 | 9.6 | 5.3 |
| Placebo | 29.1 | 0 | 10.9 |

Figure S6.4: Cumulative ranking curve plots of SGLT2 inhibitors for PBF in range network. Higher surface under the curve reflects higher probability of association with PBF.

Table S6.4: SUCRA of the effects of various SGLT2 inhibitors on PBF.

| Treatment | SUCRA | PrBest | MeanRank |
| --- | --- | --- | --- |
| Canagliflozin | 83.5 | 39.6 | 2.3 |
| Dapagliflozin | 71.5 | 6.5 | 3.3 |
| Empagliflozin | 54.7 | 0.2 | 4.6 |
| Glimepiride | 12.1 | 0 | 8 |
| Ipragliflozin | 89.6 | 53.3 | 1.8 |
| Liraglutide | 44.1 | 0.4 | 5.5 |
| Sitagliptin | 48.6 | 0 | 5.1 |
| Tofogliflozin | 23.7 | 0.1 | 7.1 |
| Placebo | 22.1 | 0 | 7.2 |

Figure S6.5: Cumulative ranking curve plots of SGLT2 inhibitors for VFA in range network. Higher SUCRA indicates a higher probability of greater reduction in VFA**.**

Table S6.5: SUCRA of the effects of various SGLT2 inhibitors on VFA.

| Treatment | SUCRA | PrBest | MeanRank |
| --- | --- | --- | --- |
| Dapagliflozin | 56.5 | 0 | 4 |
| Empagliflozin | 56 | 4.2 | 4.1 |
| Glimepiride | 12.4 | 0 | 7.1 |
| Ipragliflozin | 97.9 | 85.9 | 1.1 |
| Metformin | 52.4 | 0 | 4.3 |
| Pioglitazone | 21.3 | 0.1 | 6.5 |
| Sitagliptin | 82.1 | 9.7 | 2.3 |
| Placebo | 21.4 | 0 | 6.5 |

Figure S6.6: Cumulative ranking curve plots of SGLT2 inhibitors for SFA in range network. Higher SUCRA indicates a higher probability of greater reduction in SFA.

Table S6.6: SUCRA of the effects of various SGLT2 inhibitors on SFA.

| Treatment | SUCRA | PrBest | MeanRank |
| --- | --- | --- | --- |
| Dapagliflozin | 99.9 | 99.8 | 1 |
| Glimepiride | 67.2 | 0.2 | 2.3 |
| Ipragliflozin | 55.7 | 0.1 | 2.8 |
| Metformin | 0.5 | 0 | 5 |
| Placebo | 26.7 | 0 | 3.9 |

Figure S6.7: Cumulative ranking curve plots of SGLT2 inhibitors for BW in range network. Higher surface under the curve reflects higher probability of association with BW.

Table S6.7: SUCRA of the effects of various SGLT2 inhibitors on BW.

| Treatment | SUCRA | PrBest | MeanRank |
| --- | --- | --- | --- |
| Canagliflozin | 94.7 | 53.4 | 1.6 |
| Dapagliflozin | 75.6 | 2.6 | 3.9 |
| Empagliflozin | 77.8 | 3.5 | 3.7 |
| Glibenclamide | 5.9 | 0 | 12.3 |
| Glimepiride | 4.2 | 0 | 12.5 |
| Ipragliflozin | 69.2 | 0.6 | 4.7 |
| Linagliptin | 35.8 | 0 | 8.7 |
| Liraglutide | 70.5 | 7.4 | 4.5 |
| Loxenatide | 60.2 | 31.6 | 5.8 |
| Metformin | 36.7 | 0 | 8.6 |
| Sitagliptin | 29 | 0 | 9.5 |
| Tofogliflozin | 65.6 | 0.9 | 5.1 |
| Placebo | 24.8 | 0 | 10 |

Figure S6.8: Cumulative ranking curve plots of SGLT2 inhibitors for BMI in range network. Higher surface under the curve reflects higher probability of association with BMI.

Table S6.8: SUCRA of the effects of various SGLT2 inhibitors on BMI.

| Treatment | SUCRA | PrBest | MeanRank |
| --- | --- | --- | --- |
| Canagliflozin | 91.3 | 51.5 | 2 |
| Dapagliflozin | 73.4 | 2.5 | 4.2 |
| Empagliflozin | 77.9 | 25.8 | 3.7 |
| Glibenclamide | 13.6 | 0 | 11.4 |
| Glimepiride | 14.1 | 0 | 11.3 |
| Insulin | 0 | 0 | 13 |
| Ipragliflozin | 66.9 | 2 | 5 |
| Liraglutide | 70.2 | 9.8 | 4.6 |
| Loxenatide | 62.6 | 7.7 | 5.5 |
| Metformin | 38.1 | 0 | 8.4 |
| Sitagliptin | 45 | 0.1 | 7.6 |
| Tofogliflozin | 67.3 | 0.6 | 4.9 |
| Placebo | 29.5 | 0 | 9.5 |

Figure S6.9: Cumulative ranking curve plots of SGLT2 inhibitors for WC in range network. Higher surface under the curve reflects higher probability of association with WC.

Table S6.9: SUCRA of the effects of various SGLT2 inhibitors on WC.

| Treatment | SUCRA | PrBest | MeanRank |
| --- | --- | --- | --- |
| Canagliflozin | 69.5 | 0.1 | 4.1 |
| Dapagliflozin | 50.5 | 0 | 6 |
| Empagliflozin | 52 | 1.9 | 5.8 |
| Glimepiride | 15.2 | 0 | 9.5 |
| Ipragliflozin | 70.4 | 2.4 | 4 |
| Loxenatide | 48.4 | 3.2 | 6.2 |
| Metformin | 26.7 | 0 | 8.3 |
| Semaglutide | 89.7 | 30.5 | 2 |
| Sitagliptin | 27.9 | 0.5 | 8.2 |
| Tofogliflozin | 94.2 | 61.4 | 1.6 |
| Placebo | 5.6 | 0 | 10.4 |

Figure S6.10: Cumulative ranking curve plots of SGLT2 inhibitors for TBW in range network. Higher surface under the curve reflects higher probability of association with TBW.

Table S6.10: SUCRA of the effects of various SGLT2 inhibitors on TBW.

| Treatment | SUCRA | PrBest | MeanRank |
| --- | --- | --- | --- |
| Dapagliflozin | 44.8 | 20 | 2.7 |
| Empagliflozin | 20.9 | 1.3 | 3.4 |
| Linagliptin | 67.9 | 46.4 | 2 |
| Placebo | 66.3 | 32.3 | 2 |

Figure S6.11: Cumulative ranking probability plots of SGLT2 inhibitors for any adverse events in the network meta-analysis. A higher surface under the cumulative ranking curve (SUCRA) indicates a higher probability of being associated with increased risk of any AE.

Table S6.11: Surface under the cumulative ranking curve (SUCRA) values for the risk of any AE across SGLT2 inhibitors in the network meta-analysis.

| Treatment | SUCRA | PrBest | MeanRank |
| --- | --- | --- | --- |
| Canagliflozin | 42.4 | 0.1 | 6.8 |
| Dapagliflozin | 30 | 0.1 | 8 |
| Empagliflozin | 49.8 | 0.5 | 6 |
| Glimepiride | 27.7 | 0 | 8.2 |
| Ipragliflozin | 66.4 | 0.1 | 4.4 |
| Loxenatide | 1.2 | 0 | 10.9 |
| Metformin | 85.2 | 41.8 | 2.5 |
| Pioglitazone | 54.7 | 8.6 | 5.5 |
| Sitagliptin | 92.2 | 44.1 | 1.8 |
| Tofogliflozin | 41.3 | 4.2 | 6.9 |
| Placebo | 58.9 | 0.5 | 5.1 |

Figure S6.12: Cumulative ranking probability plots of SGLT2 inhibitors for any serious AE in the network meta-analysis. A higher surface under the cumulative ranking curve (SUCRA) indicates a higher probability of being associated with increased risk of any serious AE.

Table S6.12: Surface under the cumulative ranking curve (SUCRA) values for the risk of any serious AE across SGLT2 inhibitors in the network meta-analysis.

| Treatment | SUCRA | PrBest | MeanRank |
| --- | --- | --- | --- |
| Canagliflozin | 70.2 | 25.2 | 2.5 |
| Dapagliflozin | 34.7 | 2.1 | 4.3 |
| Empagliflozin | 59.4 | 23.5 | 3 |
| Glimepiride | 34 | 0.1 | 4.3 |
| Loxenatide | 54.7 | 43.6 | 3.3 |
| Placebo | 46.9 | 5.5 | 3.7 |

Figure S6.13: Cumulative ranking probability plots of SGLT2 inhibitors for AE leading to discontinuation in the network meta-analysis. A higher surface under the cumulative ranking curve (SUCRA) indicates a higher probability of being associated with increased risk of AE leading to discontinuation.

Table S6.13: Surface under the cumulative ranking curve (SUCRA) values for the risk of AE leading to discontinuation across SGLT2 inhibitors in the network meta-analysis.

| Treatment | SUCRA | PrBest | MeanRank |
| --- | --- | --- | --- |
| Canagliflozin | 49.8 | 3.7 | 3.5 |
| Dapagliflozin | 13.1 | 0.6 | 5.3 |
| Empagliflozin | 61 | 25.2 | 3 |
| Glimepiride | 54.8 | 9.2 | 3.3 |
| Sitagliptin | 53.1 | 41 | 3.3 |
| Placebo | 68.3 | 20.4 | 2.6 |

Figure S6.14: Cumulative ranking probability plots of SGLT2 inhibitors for genitourinary tract infection in the network meta-analysis. A higher surface under the cumulative ranking curve (SUCRA) indicates a higher probability of being associated with increased risk of genitourinary tract infection.

Table S6.14: Surface under the cumulative ranking curve (SUCRA) values for the risk of genitourinary tract infection across SGLT2 inhibitors in the network meta-analysis.

| Treatment | SUCRA | PrBest | MeanRank |
| --- | --- | --- | --- |
| Canagliflozin | 60.6 | 0 | 4.5 |
| Dapagliflozin | 43 | 0.2 | 6.1 |
| Empagliflozin | 37.2 | 2.6 | 6.6 |
| Glimepiride | 83 | 4.1 | 2.5 |
| Ipragliflozin | 19.5 | 0 | 8.2 |
| Loxenatide | 59.4 | 19.8 | 4.7 |
| Metformin | 2.7 | 0 | 9.8 |
| Semaglutide | 95.1 | 66.9 | 1.4 |
| Sitagliptin | 50.5 | 6 | 5.5 |
| Placebo | 49 | 0.4 | 5.6 |

**Appendix 7: league tables of summary estimates for SGLT2 inhibitorsderived from network meta-analysis of 33trials**

**Table S7.1:** SMM

Each cell presents the mean difference in SMM between the row-defining intervention and the column-defining intervention. The rows represent the comparison of the row drug class to the columndrug class. The effect estimates are expressed as mean difference (95% confidence interval). A mean difference <0 favors the drug in the column, while a mean difference >0 favors thedrug in the row.

| Canagliflozin |  |  |  |  |  |  |
| --- | --- | --- | --- | --- | --- | --- |
| -1.70 (-14.70,11.30) | Dapagliflozin |  |  |  |  |  |
| -1.34 (-14.36,11.68) | 0.36  (-0.72,1.45) | Empagliflozin |  |  |  |  |
| -1.43 (-15.48,12.63) | 0.27  (-5.15,5.69) | -0.09  (-5.57,5.38) | Ipragliflozin |  |  |  |
| -2.41 (-15.48,10.66) | -0.71  (-2.24,0.83) | -1.07  (-2.15,0.01) | -0.98  (-6.56,4.60) | Linagliptin |  |  |
| -0.00  (-4.25,4.25) | 1.70 (-11.98,15.38) | 1.34 (-12.36,15.04) | 1.43 (-13.26,16.11) | 2.41 (-11.33,16.15) | Loxenatide |  |
| -2.02 (-15.01,10.97) | -0.32  (-0.85,0.21) | -0.68  (-1.63,0.26) | -0.59  (-5.98,4.80) | 0.39  (-1.05,1.83) | -2.02 (-15.69,11.65) | Placebo |

**Table S7.2: LM**

Each cell presents the mean difference in LM between the row-defining intervention and the column-defining intervention. The rows represent the comparison of the row drug class to the columndrug class. The effect estimates are expressed as mean difference (95% confidence interval). Amean difference <0 favors the drug in the column, while a mean difference >0 favors thedrug in the row.

| Canagliflozin |  |  |  |  |  |  |  |  |  |  |  |
| --- | --- | --- | --- | --- | --- | --- | --- | --- | --- | --- | --- |
| -0.67 (-1.37,0.03) | Dapagliflozin |  |  |  |  |  |  |  |  |  |  |
| 0.42 (-0.35,1.19) | 1.08 (0.36,1.81) | Empagliflozin |  |  |  |  |  |  |  |  |  |
| -1.94 (-2.88,-1.00) | -1.28 (-1.90,-0.65) | -2.36 (-3.32,-1.40) | Glibenclamide |  |  |  |  |  |  |  |  |
| -2.05 (-2.64,-1.46) | -1.39 (-2.11,-0.66) | -2.47 (-3.34,-1.60) | -0.11 (-1.07,0.85) | Glimepiride |  |  |  |  |  |  |  |
| -0.87 (-3.38,1.64) | -0.20 (-2.61,2.21) | -1.29 (-3.80,1.23) | 1.07 (-1.42,3.56) | 1.18 (-1.33,3.70) | Insulin |  |  |  |  |  |  |
| 0.19 (-0.55,0.94) | 0.86 (0.16,1.56) | -0.22 (-0.90,0.45) | 2.14 (1.20,3.07) | 2.25 (1.40,3.10) | 1.06 (-1.44,3.57) | Ipragliflozin |  |  |  |  |  |
| 0.32 (-1.00,1.63) | 0.98 (-0.30,2.27) | -0.10 (-1.17,0.97) | 2.26 (0.83,3.69) | 2.37 (0.99,3.75) | 1.19 (-1.54,3.92) | 0.12 (-1.14,1.39) | Liraglutide |  |  |  |  |
| 0.78 (-0.13,1.69) | 1.45 (0.30,2.59) | 0.36 (-0.83,1.55) | 2.72 (1.42,4.03) | 2.83 (1.75,3.91) | 1.65 (-1.02,4.32) | 0.59 (-0.59,1.76) | 0.46 (-1.14,2.06) | Semaglutide |  |  |  |
| -0.11 (-3.57,3.36) | 0.56 (-2.89,4.01) | -0.52 (-3.97,2.92) | 1.84 (-1.67,5.35) | 1.95 (-1.54,5.43) | 0.76 (-3.45,4.97) | -0.30 (-3.68,3.08) | -0.42 (-4.03,3.19) | -0.89 (-4.46,2.69) | Sitagliptin |  |  |
| 0.15 (-0.97,1.27) | 0.81 (-0.38,2.01) | -0.27 (-1.56,1.02) | 2.09 (0.74,3.44) | 2.20 (1.25,3.15) | 1.02 (-1.67,3.71) | -0.05 (-1.32,1.23) | -0.17 (-1.84,1.51) | -0.63 (-2.07,0.81) | 0.25 (-3.36,3.87) | Tofogliflozin |  |
| -0.80 (-1.39,-0.21) | -0.13 (-0.66,0.39) | -1.22 (-1.71,-0.72) | 1.14 (0.32,1.96) | 1.25 (0.54,1.97) | 0.07 (-2.40,2.54) | -0.99 (-1.45,-0.54) | -1.12 (-2.29,0.06) | -1.58 (-2.66,-0.50) | -0.69 (-4.11,2.72) | -0.95 (-2.14,0.24) | Placebo |

**Table S7.3: FM**

Each cell presents the mean difference in FM between the row-defining intervention and the column-defining intervention. The rows represent the comparison of the row drug class to the columndrug class. The effect estimates are expressed as mean difference (95% confidence interval). Amean difference <0 favors the drug in the column, while a mean difference >0 favors thedrug in the row.

| Canagliflozin |  |  |  |  |  |  |  |  |  |  |  |  |  |  |
| --- | --- | --- | --- | --- | --- | --- | --- | --- | --- | --- | --- | --- | --- | --- |
| 0.41 (-0.58,1.41) | Dapagliflozin |  |  |  |  |  |  |  |  |  |  |  |  |  |
| -0.07 (-1.18,1.05) | -0.48 (-1.64,0.68) | Empagliflozin |  |  |  |  |  |  |  |  |  |  |  |  |
| -1.96 (-3.44,-0.48) | -2.37 (-3.47,-1.27) | -1.89 (-3.49,-0.29) | Glibenclamide |  |  |  |  |  |  |  |  |  |  |  |
| -1.34 (-2.04,-0.64) | -1.75 (-2.70,-0.80) | -1.27 (-2.49,-0.05) | 0.62 (-0.83,2.07) | Glimepiride |  |  |  |  |  |  |  |  |  |  |
| -5.71 (-8.89,-2.52) | -6.12 (-9.14,-3.09) | -5.64 (-8.88,-2.40) | -3.75 (-6.96,-0.53) | -4.37 (-7.54,-1.20) | Insulin |  |  |  |  |  |  |  |  |  |
| 0.38 (-1.02,1.77) | -0.03 (-1.48,1.41) | 0.45 (-0.94,1.84) | 2.34 (0.52,4.16) | 1.72 (0.22,3.21) | 6.08 (2.73,9.44) | Ipragliflozin |  |  |  |  |  |  |  |  |
| -0.73 (-2.62,1.16) | -1.14 (-3.06,0.77) | -0.66 (-2.18,0.86) | 1.23 (-0.98,3.44) | 0.61 (-1.34,2.56) | 4.98 (1.40,8.55) | -1.11 (-3.17,0.95) | Linagliptin |  |  |  |  |  |  |  |
| -0.17 (-1.74,1.41) | -0.58 (-2.19,1.02) | -0.10 (-1.21,1.01) | 1.79 (-0.16,3.74) | 1.17 (-0.48,2.82) | 5.54 (2.11,8.96) | -0.55 (-2.33,1.23) | 0.56 (-1.32,2.44) | Liraglutide |  |  |  |  |  |  |
| -0.70 (-2.99,1.59) | -1.11 (-3.61,1.38) | -0.63 (-3.18,1.91) | 1.26 (-1.47,3.98) | 0.64 (-1.75,3.03) | 5.01 (1.09,8.92) | -1.08 (-3.76,1.60) | 0.03 (-2.94,2.99) | -0.53 (-3.31,2.24) | Loxenatide |  |  |  |  |  |
| -2.07 (-3.93,-0.20) | -2.48 (-4.37,-0.59) | -2.00 (-3.65,-0.35) | -0.11 (-2.30,2.08) | -0.73 (-2.66,1.20) | 3.64 (0.07,7.20) | -2.45 (-4.48,-0.41) | -1.34 (-3.58,0.91) | -1.90 (-3.89,0.09) | -1.37 (-4.32,1.58) | Pioglitazone |  |  |  |  |
| 0.79 (-0.85,2.43) | 0.38 (-1.54,2.30) | 0.86 (-1.13,2.85) | 2.75 (0.54,4.96) | 2.13 (0.34,3.91) | 6.50 (2.91,10.08) | 0.41 (-1.74,2.57) | 1.52 (-0.98,4.02) | 0.96 (-1.32,3.23) | 1.49 (-1.33,4.31) | 2.86 (0.37,5.34) | Semaglutide |  |  |  |
| -0.82 (-3.59,1.94) | -1.23 (-4.03,1.56) | -0.75 (-3.52,2.01) | 1.14 (-1.87,4.14) | 0.52 (-2.30,3.33) | 4.88 (0.77,9.00) | -1.20 (-3.59,1.19) | -0.09 (-3.25,3.06) | -0.65 (-3.63,2.33) | -0.12 (-3.71,3.47) | 1.25 (-1.89,4.39) | -1.61 (-4.83,1.60) | Sitagliptin |  |  |
| 0.06 (-1.39,1.52) | -0.35 (-1.94,1.24) | 0.13 (-1.63,1.90) | 2.02 (0.09,3.95) | 1.40 (0.13,2.67) | 5.77 (2.35,9.18) | -0.32 (-2.28,1.65) | 0.79 (-1.54,3.12) | 0.23 (-1.86,2.32) | 0.76 (-1.95,3.47) | 2.13 (-0.18,4.44) | -0.73 (-2.92,1.47) | 0.88 (-2.21,3.97) | Tofogliflozin |  |
| -1.34 (-2.13,-0.54) | -1.75 (-2.63,-0.87) | -1.27 (-2.06,-0.48) | 0.62 (-0.79,2.03) | 0.00 (-0.95,0.95) | 4.37 (1.22,7.52) | -1.72 (-2.86,-0.57) | -0.61 (-2.32,1.11) | -1.17 (-2.53,0.19) | -0.64 (-3.06,1.78) | 0.73 (-0.96,2.42) | -2.13 (-3.95,-0.30) | -0.52 (-3.16,2.13) | -1.40 (-2.99,0.19) | Placebo |

**Table S7.4: PBF**

Each cell presents the mean difference in PBF between the row-defining intervention and the column-defining intervention. The rows represent the comparison of the row drug class to the columndrug class. The effect estimates are expressed as mean difference (95% confidence interval). Amean difference <0 favors the drug in the column, while a mean difference >0 favors thedrug in the row.

| Canagliflozin |  |  |  |  |  |  |  |  |
| --- | --- | --- | --- | --- | --- | --- | --- | --- |
| -0.48  (-1.81,0.84) | Dapagliflozin |  |  |  |  |  |  |  |
| -1.06  (-3.29,1.18) | -0.57  (-2.37,1.23) | Empagliflozin |  |  |  |  |  |  |
| -2.42 (-3.26,-1.59) | -1.94 (-2.97,-0.91) | -1.37 (-3.44,0.70) | Glimepiride |  |  |  |  |  |
| 0.15  (-2.36,2.67) | 0.64  (-1.50,2.78) | 1.21  (0.05,2.37) | 2.58  (0.20,4.95) | Ipragliflozin |  |  |  |  |
| -1.26  (-3.60,1.09) | -0.77  (-2.70,1.16) | -0.20 (-0.91,0.51) | 1.17 (-1.02,3.36) | -1.41 (-2.77,-0.05) | Liraglutide |  |  |  |
| -1.16  (-3.56,1.24) | -0.67  (-2.68,1.33) | -0.10 (-0.98,0.78) | 1.27 (-0.98,3.52) | -1.31 (-2.06,-0.56) | 0.10  (-1.03,1.23) | Sitagliptin |  |  |
| -2.09 (-3.61,-0.58) | -1.61  (-3.24,0.02) | -1.04 (-3.47,1.39) | 0.33 (-0.94,1.60) | -2.25  (-4.94,0.44) | -0.84 (-3.37,1.69) | -0.94 (-3.52,1.65) | Tofogliflozin |  |
| -1.93 (-3.72,-0.14) | -1.44 (-2.65,-0.24) | -0.87 (-2.21,0.47) | 0.50 (-1.09,2.08) | -2.08 (-3.85,-0.31) | -0.67 (-2.19,0.84) | -0.77 (-2.38,0.83) | 0.17 (-1.86,2.19) | Placebo |

**Table S7.5: VFA**

Each cell presents the mean difference in VFA between the row-defining intervention and the column-defining intervention. The rows represent the comparison of the row drug class to the columndrug class. The effect estimates are expressed as mean difference (95% confidence interval). Amean difference <0 favors the drug in the column, while a mean difference >0 favors thedrug in the row.

| Dapagliflozin |  |  |  |  |  |  |  |
| --- | --- | --- | --- | --- | --- | --- | --- |
| 0.23 (-24.98,25.43) | Empagliflozin |  |  |  |  |  |  |
| -17.54 (-32.66,-2.42) | -17.77 (-47.16,11.63) | Glimepiride |  |  |  |  |  |
| 21.11 (11.30,30.91) | 20.88 (-3.09,44.85) | 38.65 (20.62,56.67) | Ipragliflozin |  |  |  |  |
| -1.21 (-16.18,13.76) | -1.44 (-27.95,25.07) | 16.33 (-4.95,37.61) | -22.32 (-33.63,-11.01) | Metformin |  |  |  |
| -14.07 (-39.43,11.29) | -14.30 (-34.91,6.31) | 3.47 (-26.06,33.00) | -35.18 (-59.30,-11.06) | -12.86 (-39.50,13.78) | Pioglitazone |  |  |
| 12.61 (-3.56,28.78) | 12.38 (-14.82,39.58) | 30.15 (8.01,52.29) | -8.50  (-21.36,4.36) | 13.82 (-3.31,30.94) | 26.68 (-0.65,54.01) | Sitagliptin |  |
| -11.97 (-20.90,-3.04) | -12.20 (-35.82,11.42) | 5.57 (-11.99,23.13) | -33.08 (-37.18,-28.97) | -10.76 (-22.79,1.28) | 2.10 (-21.67,25.87) | -24.58 (-38.07,-11.08) | Placebo |

**Table S7.6: SFA**

Each cell presents the mean difference in SFA between the row-defining intervention and the column-defining intervention. The rows represent the comparison of the row drug class to the columndrug class. The effect estimates are expressed as mean difference (95% confidence interval). Amean difference <0 favors the drug in the column, while a mean difference >0 favors thedrug in the row.

| Dapagliflozin |  |  |  |  |
| --- | --- | --- | --- | --- |
| -18.39  (-30.31,-6.47) | Glimepiride |  |  |  |
| -27.05  (-46.04,-8.05) | -8.66  (-31.08,13.77) | Ipragliflozin |  |  |
| -47.19  (-68.61,-25.77) | -28.80  (-53.31,-4.29) | -20.14  (-30.04,-10.24) | Metformin |  |
| -35.02  (-53.69,-16.34) | -16.63  (-38.78,5.53) | -7.97  (-11.61,-4.33) | 12.17  (1.62,22.72) | Placebo |

**Table S7.7: BW**

Each cell presents the mean difference in BW between the row-defining intervention and the column-defining intervention. The rows represent the comparison of the row drug class to the columndrug class. The effect estimates are expressed as mean difference (95% confidence interval). Amean difference <0 favors the drug in the column, while a mean difference >0 favors thedrug in the row.

| Canagliflozin |  |  |  |  |  |  |  |  |  |  |  |  |
| --- | --- | --- | --- | --- | --- | --- | --- | --- | --- | --- | --- | --- |
| -0.63 (-1.37,0.12) | Dapagliflozin |  |  |  |  |  |  |  |  |  |  |  |
| -0.59 (-1.46,0.29) | 0.04 (-0.79,0.87) | Empagliflozin |  |  |  |  |  |  |  |  |  |  |
| -4.43 (-5.73,-3.12) | -3.80 (-4.87,-2.73) | -3.84 (-5.20,-2.49) | Glibenclamide |  |  |  |  |  |  |  |  |  |
| -4.55 (-4.92,-4.19) | -3.93 (-4.67,-3.19) | -3.97 (-4.87,-3.07) | -0.13 (-1.43,1.18) | Glimepiride |  |  |  |  |  |  |  |  |
| -0.79 (-1.53,-0.05) | -0.16 (-0.85,0.53) | -0.20 (-0.83,0.43) | 3.64 (2.36,4.92) | 3.77 (2.99,4.54) | Ipragliflozin |  |  |  |  |  |  |  |
| -2.44 (-3.59,-1.29) | -1.81 (-2.93,-0.69) | -1.85 (-2.60,-1.10) | 1.99 (0.44,3.54) | 2.12 (0.95,3.29) | -1.65 (-2.63,-0.67) | Linagliptin |  |  |  |  |  |  |
| -0.79 (-2.10,0.53) | -0.16 (-1.44,1.12) | -0.20 (-1.18,0.78) | 3.64 (1.97,5.32) | 3.77 (2.44,5.10) | 0.00 (-1.16,1.17) | 1.65 (0.42,2.88) | Liraglutide |  |  |  |  |  |
| -1.10 (-5.93,3.73) | -0.47 (-5.36,4.41) | -0.51 (-5.42,4.39) | 3.33 (-1.67,8.33) | 3.45 (-1.39,8.29) | -0.31 (-5.19,4.57) | 1.34 (-3.63,6.30) | -0.31 (-5.32,4.69) | Loxenatide |  |  |  |  |
| -2.41 (-3.48,-1.35) | -1.79 (-2.82,-0.76) | -1.83 (-2.82,-0.84) | 2.01 (0.53,3.50) | 2.14 (1.05,3.23) | -1.63 (-2.39,-0.86) | 0.02 (-1.22,1.26) | -1.63 (-3.02,-0.24) | -1.31 (-6.26,3.63) | Metformin |  |  |  |
| -2.71 (-3.63,-1.80) | -2.09 (-2.96,-1.21) | -2.13 (-2.90,-1.36) | 1.72 (0.33,3.10) | 1.84 (0.90,2.78) | -1.92 (-2.48,-1.37) | -0.28 (-1.35,0.80) | -1.93 (-3.17,-0.68) | -1.61 (-6.52,3.30) | -0.30 (-1.24,0.65) | Sitagliptin |  |  |
| -0.95 (-1.86,-0.05) | -0.33 (-1.44,0.78) | -0.37 (-1.59,0.86) | 3.47 (1.93,5.02) | 3.60 (2.77,4.43) | -0.17 (-1.30,0.97) | 1.48 (0.05,2.92) | -0.17 (-1.74,1.40) | 0.15 (-4.76,5.06) | 1.46 (0.09,2.83) | 1.76 (0.50,3.01) | Tofogliflozin |  |
| -2.82 (-3.48,-2.15) | -2.19 (-2.80,-1.58) | -2.23 (-2.80,-1.67) | 1.61 (0.38,2.84) | 1.74 (1.03,2.44) | -2.03 (-2.36,-1.70) | -0.38 (-1.32,0.56) | -2.03 (-3.16,-0.90) | -1.72 (-6.59,3.15) | -0.40 (-1.23,0.43) | -0.11 (-0.73,0.52) | -1.86 (-2.95,-0.78) | Placebo |

**Table S7.8: BMI**

Each cell presents the mean difference in BMI between the row-defining intervention and the column-defining intervention. The rows represent the comparison of the row drug class to the columndrug class. The effect estimates are expressed as mean difference (95% confidence interval). Amean difference <0 favors the drug in the column, while a mean difference >0 favors thedrug in the row.

| Canagliflozin |  |  |  |  |  |  |  |  |  |  |  |  |
| --- | --- | --- | --- | --- | --- | --- | --- | --- | --- | --- | --- | --- |
| -0.31 (-0.73,0.11) | Dapagliflozin |  |  |  |  |  |  |  |  |  |  |  |
| -0.21 (-1.52,1.09) | 0.10 (-1.14,1.34) | Empagliflozin |  |  |  |  |  |  |  |  |  |  |
| -1.71 (-2.28,-1.15) | -1.40 (-1.78,-1.02) | -1.50 (-2.79,-0.20) | Glibenclamide |  |  |  |  |  |  |  |  |  |
| -1.70 (-1.89,-1.50) | -1.38 (-1.75,-1.02) | -1.48 (-2.77,-0.19) | 0.02 (-0.51,0.54) | Glimepiride |  |  |  |  |  |  |  |  |
| -5.01 (-7.26,-2.76) | -4.70 (-6.91,-2.49) | -4.80 (-7.34,-2.26) | -3.30 (-5.55,-1.05) | -3.32 (-5.56,-1.07) | Insulin |  |  |  |  |  |  |  |
| -0.41 (-0.97,0.15) | -0.10 (-0.48,0.27) | -0.20 (-1.38,0.98) | 1.30 (0.77,1.83) | 1.28 (0.76,1.81) | 4.60 (2.35,6.84) | Ipragliflozin |  |  |  |  |  |  |
| -0.31 (-1.65,1.02) | -0.00 (-1.27,1.27) | -0.10 (-0.38,0.18) | 1.40 (0.07,2.72) | 1.38 (0.06,2.70) | 4.70 (2.15,7.25) | 0.10 (-1.11,1.31) | Liraglutide |  |  |  |  |  |
| -0.50 (-1.29,0.29) | -0.19 (-1.09,0.71) | -0.29 (-1.82,1.24) | 1.21 (0.24,2.19) | 1.20 (0.38,2.01) | 4.51 (2.12,6.90) | -0.09 (-1.06,0.88) | -0.19 (-1.74,1.37) | Loxenatide |  |  |  |  |
| -1.05 (-1.68,-0.42) | -0.74 (-1.21,-0.26) | -0.83 (-2.05,0.38) | 0.66 (0.06,1.27) | 0.65 (0.05,1.25) | 3.96 (1.70,6.23) | -0.63 (-0.93,-0.34) | -0.73 (-1.98,0.51) | -0.55 (-1.56,0.47) | Metformin |  |  |  |
| -0.81 (-2.01,0.38) | -0.50 (-1.63,0.62) | -0.60 (-1.12,-0.08) | 0.90 (-0.29,2.08) | 0.88 (-0.30,2.07) | 4.20 (1.71,6.68) | -0.40 (-1.46,0.66) | -0.50 (-1.09,0.09) | -0.31 (-1.75,1.12) | 0.23 (-0.87,1.33) | Sitagliptin |  |  |
| -0.40 (-0.76,-0.04) | -0.08 (-0.56,0.39) | -0.18 (-1.51,1.14) | 1.32 (0.71,1.92) | 1.30 (1.00,1.60) | 4.62 (2.35,6.88) | 0.02 (-0.59,0.62) | -0.08 (-1.44,1.27) | 0.10 (-0.77,0.98) | 0.65 (-0.02,1.32) | 0.42 (-0.80,1.64) | Tofogliflozin |  |
| -1.21 (-1.76,-0.67) | -0.90 (-1.26,-0.54) | -1.00 (-2.19,0.19) | 0.50 (-0.02,1.02) | 0.48 (-0.03,1.00) | 3.80 (1.56,6.04) | -0.80 (-0.91,-0.69) | -0.90 (-2.12,0.32) | -0.71 (-1.68,0.25) | -0.17 (-0.48,0.15) | -0.40 (-1.47,0.67) | -0.82 (-1.41,-0.22) | Placebo |

**Table S7.9: WC**

Each cell presents the mean difference in WC between the row-defining intervention and the column-defining intervention. The rows represent the comparison of the row drug class to the columndrug class. The effect estimates are expressed as mean difference (95% confidence interval). Amean difference <0 favors the drug in the column, while a mean difference >0 favors thedrug in the row.

| Canagliflozin |  |  |  |  |  |  |  |  |  |  |
| --- | --- | --- | --- | --- | --- | --- | --- | --- | --- | --- |
| -1.03 (-2.68,0.62) | Dapagliflozin |  |  |  |  |  |  |  |  |  |
| -1.00 (-3.85,1.84) | 0.03 (-2.29,2.34) | Empagliflozin |  |  |  |  |  |  |  |  |
| -3.24 (-3.83,-2.65) | -2.21 (-3.75,-0.67) | -2.24 (-5.02,0.54) | Glimepiride |  |  |  |  |  |  |  |
| -0.12 (-2.16,1.93) | 0.91 (-0.30,2.12) | 0.89 (-1.19,2.96) | 3.12 (1.16,5.08) | Ipragliflozin |  |  |  |  |  |  |
| -1.20 (-4.63,2.23) | -0.17 (-3.98,3.64) | -0.20 (-4.65,4.26) | 2.04 (-1.44,5.52) | -1.08 (-5.08,2.91) | Loxenatide |  |  |  |  |  |
| -2.50 (-4.83,-0.17) | -1.47 (-3.11,0.17) | -1.50 (-3.85,0.86) | 0.74 (-1.51,2.99) | -2.38 (-3.49,-1.28) | -1.30 (-5.44,2.85) | Metformin |  |  |  |  |
| 1.40 (-0.23,3.03) | 2.43 (0.11,4.75) | 2.40 (-0.87,5.68) | 4.64 (2.91,6.37) | 1.52 (-1.10,4.13) | 2.60 (-1.20,6.40) | 3.90 (1.06,6.74) | Semaglutide |  |  |  |
| -2.52 (-5.93,0.89) | -1.49 (-4.47,1.50) | -1.51 (-4.94,1.91) | 0.72 (-2.64,4.08) | -2.40 (-5.13,0.33) | -1.32 (-6.15,3.52) | -0.02 (-2.96,2.92) | -3.92 (-7.70,-0.14) | Sitagliptin |  |  |
| 1.96 (-0.15,4.07) | 2.99 (0.44,5.54) | 2.96 (-0.48,6.41) | 5.20 (3.17,7.23) | 2.08 (-0.74,4.90) | 3.16 (-0.87,7.19) | 4.46 (1.43,7.49) | 0.56 (-2.11,3.23) | 4.48 (0.55,8.40) | Tofogliflozin |  |
| -3.82 (-5.81,-1.82) | -2.79 (-3.91,-1.67) | -2.81 (-4.84,-0.79) | -0.58 (-2.48,1.33) | -3.70 (-4.16,-3.24) | -2.62 (-6.59,1.35) | -1.32 (-2.52,-0.12) | -5.22 (-7.79,-2.64) | -1.30 (-4.06,1.46) | -5.78 (-8.56,-2.99) | Placebo |

**Table S7.10: TBW**

Each cell presents the mean difference in TBW between the row-defining intervention and the column-defining intervention. The rows represent the comparison of the row drug class to the columndrug class. The effect estimates are expressed as mean difference (95% confidence interval). A mean difference <0 favors the drug in the column, while a mean difference >0 favors thedrug in the row.

| Dapagliflozin |  |  |  |
| --- | --- | --- | --- |
| 0.41 (-1.53,2.34) | Empagliflozin |  |  |
| -0.44 (-2.90,2.02) | -0.85 (-2.37,0.67) | Linagliptin |  |
| -0.35 (-1.97,1.28) | -0.76 (-2.18,0.67) | 0.09 (-1.99,2.18) | Placebo |

**Table S7.11: Any AE**

Each cell presents the mean difference in any AE between the row-defining intervention and the column-defining intervention. The rows represent the comparison of the row drug class to the columndrug class. The effect estimates are expressed as mean difference (95% confidence interval). Amean difference <0 favors the drug in the column, while a mean difference >0 favors thedrug in the row.

| Canagliflozin |  |  |  |  |  |  |  |  |  |  |
| --- | --- | --- | --- | --- | --- | --- | --- | --- | --- | --- |
| 0.87 (0.54,1.41) | Dapagliflozin |  |  |  |  |  |  |  |  |  |
| 1.16 (0.60,2.22) | 1.33 (0.66,2.69) | Empagliflozin |  |  |  |  |  |  |  |  |
| 0.89 (0.77,1.02) | 1.02 (0.63,1.66) | 0.77 (0.39,1.49) | Glimepiride |  |  |  |  |  |  |  |
| 1.85 (0.52,6.58) | 2.13 (0.59,7.78) | 1.60 (0.47,5.47) | 2.09 (0.59,7.47) | Ipragliflozin |  |  |  |  |  |  |
| 0.08 (0.01,0.71) | 0.09 (0.01,0.87) | 0.07 (0.01,0.68) | 0.09 (0.01,0.81) | 0.04 (0.00,0.54) | Loxenatide |  |  |  |  |  |
| 3.60 (0.57,22.80) | 4.14 (0.64,26.75) | 3.11 (0.50,19.20) | 4.06 (0.64,25.85) | 1.94 (0.51,7.44) | 45.55 (2.57,805.89) | Metformin |  |  |  |  |
| 1.35 (0.30,6.00) | 1.55 (0.34,7.07) | 1.16 (0.28,4.91) | 1.52 (0.34,6.80) | 0.73 (0.11,4.64) | 17.04 (1.19,243.71) | 0.37 (0.04,3.69) | Pioglitazone |  |  |  |
| 3.83 (1.07,13.74) | 4.41 (1.20,16.25) | 3.31 (0.97,11.28) | 4.32 (1.20,15.60) | 2.07 (1.20,3.55) | 48.49 (3.80,617.90) | 1.06 (0.25,4.53) | 2.85 (0.44,18.21) | Sitagliptin |  |  |
| 0.95 (0.21,4.21) | 1.10 (0.23,5.21) | 0.82 (0.16,4.17) | 1.07 (0.24,4.73) | 0.51 (0.07,3.62) | 12.04 (0.85,171.66) | 0.26 (0.02,2.83) | 0.71 (0.09,5.82) | 0.25 (0.03,1.76) | Tofogliflozin |  |
| 1.24 (0.87,1.76) | 1.43 (0.92,2.21) | 1.07 (0.62,1.86) | 1.40 (0.97,2.03) | 0.67 (0.20,2.26) | 15.68 (1.69,145.70) | 0.34 (0.06,2.11) | 0.92 (0.22,3.93) | 0.32 (0.09,1.11) | 1.30 (0.28,6.00) | Placebo |

**Table S7.12: Any serious AE**

Each cell presents the mean difference in any serious AE between the row-defining intervention and the column-defining intervention. The rows represent the comparison of the row drug class to the columndrug class. The effect estimates are expressed as mean difference (95% confidence interval). Amean difference <0 favors the drug in the column, while a mean difference >0 favors thedrug in the row.

| Canagliflozin |  |  |  |  |  |
| --- | --- | --- | --- | --- | --- |
| 0.61  (0.17,2.11) | Dapagliflozin |  |  |  |  |
| 0.88  (0.15,5.10) | 1.45  (0.42,4.99) | Empagliflozin |  |  |  |
| 0.62  (0.43,0.89) | 1.02  (0.31,3.35) | 0.70  (0.13,3.91) | Glimepiride |  |  |
| 1.00  (0.02,52.85) | 1.65  (0.03,105.35) | 1.14  (0.01,86.94) | 1.62  (0.03,86.87) | Loxenatide |  |
| 0.71  (0.16,3.11) | 1.17  (0.54,2.57) | 0.81  (0.31,2.10) | 1.15  (0.28,4.80) | 0.71  (0.01,49.09) | Placebo |

**Table S7.13:**AE leading to discontinuation

Each cell presents the mean difference in AE leading to discontinuationbetween the row-defining intervention and the column-defining intervention. The rows represent the comparison of the row drug class to the columndrug class. The effect estimates are expressed as mean difference (95% confidence interval). Amean difference <0 favors the drug in the column, while a mean difference >0 favors thedrug in the row.

| Canagliflozin |  |  |  |  |  |
| --- | --- | --- | --- | --- | --- |
| 0.45  (0.16,1.24) | Dapagliflozin |  |  |  |  |
| 1.20  (0.21,6.75) | 2.68  (0.40,18.14) | Empagliflozin |  |  |  |
| 1.03  (0.78,1.35) | 2.29  (0.82,6.40) | 0.86  (0.15,4.88) | Glimepiride |  |  |
| 1.10  (0.01,83.25) | 2.45  (0.03,200.50) | 0.91  (0.02,48.33) | 1.07  (0.01,81.42) | Sitagliptin |  |
| 1.18  (0.72,1.96) | 2.64  (1.00,6.96) | 0.98  (0.19,5.11) | 1.15  (0.65,2.02) | 1.08  (0.01,78.90) | Placebo |

**Table S7.14:**Genitourinary tract infection

Each cell presents the mean difference in genitourinary tract infectionbetween the row-defining intervention and the column-defining intervention. The rows represent the comparison of the row drug class to the columndrug class. The effect estimates are expressed as mean difference (95% confidence interval). Amean difference <0 favors the drug in the column, while a mean difference >0 favors thedrug in the row.

| Canagliflozin |  |  |  |  |  |  |  |  |  |
| --- | --- | --- | --- | --- | --- | --- | --- | --- | --- |
| 0.37 (0.04,3.21) | Dapagliflozin |  |  |  |  |  |  |  |  |
| 0.26 (0.01,6.08) | 0.70  (0.07,6.97) | Empagliflozin |  |  |  |  |  |  |  |
| 2.96 (2.17,4.04) | 7.91 (0.94,66.28) | 11.31 (0.49,259.14) | Glimepiride |  |  |  |  |  |  |
| 0.12 (0.01,1.89) | 0.33  (0.06,1.78) | 0.47  (0.03,6.41) | 0.04 (0.00,0.63) | Ipragliflozin |  |  |  |  |  |
| 1.00 (0.02,52.85) | 2.67 (0.03,243.51) | 3.82 (0.02,605.00) | 0.34 (0.01,18.08) | 8.11 (0.07,1001.41) | Loxenatide |  |  |  |  |
| 0.02 (0.00,0.67) | 0.05  (0.00,0.85) | 0.08  (0.00,2.32) | 0.01 (0.00,0.22) | 0.16  (0.02,1.46) | 0.02 (0.00,3.99) | Metformin |  |  |  |
| 5.30 (2.64,10.64) | 14.17 (1.48,135.60) | 20.27 (0.81,508.75) | 1.79 (0.83,3.84) | 42.97 (2.57,719.50) | 5.30 (0.09,297.60) | 263.82 (7.42,9383.52) | Semaglutide |  |  |
| 0.47 (0.02,12.78) | 1.25 (0.10,15.48) | 1.78 (0.07,44.27) | 0.16 (0.01,4.26) | 3.78 (0.58,24.57) | 0.47 (0.00,81.82) | 23.22 (1.30,415.17) | 0.09 (0.00,2.59) | Sitagliptin |  |
| 0.43 (0.04,4.29) | 1.15  (0.51,2.60) | 1.65 (0.19,14.18) | 0.15 (0.01,1.42) | 3.50 (0.80,15.33) | 0.43 (0.00,42.26) | 21.48 (1.52,302.50) | 0.08 (0.01,0.90) | 0.92 (0.09,10.04) | Placebo |

**Appendix 8: CINeMA Assessment and contribution matrices**

Figure S8.1 Contribution of risk of bias across intervention groups for SMM

Figure S8.2: Overall bias assessment across treatment comparisons for SMM

Figure S8.3: Contribution of risk of bias across intervention groups for LM

Figure S8.4: Overall bias assessment across treatment comparisons for LM

Table S8.1:Transitivity (indirectness) assessment: distribution of participant characteristics

| **Intervention** | **Age (years)** | **BMI (kg/m²)** | **HbA1c (%)** | **diabetes duration (years)** | **Male (%)** | **Follow-up duration (weeks)** |
| --- | --- | --- | --- | --- | --- | --- |
| Canagliflozin | 57.8 ± 9.8 | 30.7 ± 5.1 | 8.05 ± 0.92 | 6.8 ± 5.9 | 52.2 | 48.1 |
| Dapagliflozin | 58.9 ± 10.7 | 29.4 ± 4.6 | 7.82 ± 0.85 | 7.7 ± 5.5 | 62.6 | 39.4 |
| Empagliflozin | 59.6 ± 11.2 | 28.6 ± 4.8 | 7.94 ± 0.96 | 8.8 ± 6.6 | 58.1 | 29.6 |
| Ipragliflozin | 57.2 ± 10.5 | 28.5 ± 4.7 | 7.98 ± 0.88 | 9.2 ± 6.8 | 62.9 | 22.2 |
| Tofogliflozin | 57.3 ± 11.4 | 25.3 ± 3.9 | 7.45 ± 0.45 | 10.3 ± 6.1 | 54.5 | 24 |
| Glimepiride | 56.6 ± 9.4 | 30.9 ± 5.5 | 7.90 ± 0.80 | 6.6 ± 5.0 | 54.4 | 51.2 |
| Glibenclamide | 58.0 ± 7.0 | 31.0 ± 7.0 | 7.90 ± 1.30 | NA | 60 | 12 |
| Metformin | 58.7 ± 11.5 | 28.2 ± 5.2 | 8.05 ± 0.90 | 5.3 ± 4.9 | 56.6 | 24 |
| Sitagliptin | 52.9 ± 10.6 | 28.6 ± 4.6 | 7.45 ± 0.85 | 8.6 ± 6.3 | 64.9 | 17 |
| Linagliptin | 58.8 ± 10.1 | 27.9 ± 4.2 | 9.10 ± 1.30 | 11.1 ± 6.2 | 27.5 | 24 |
| Pioglitazone | 52.5 ± 7.9 | 29.4 ± 3.7 | 8.23 ± 0.79 | 7.3 ± 5.2 | 50 | 24 |
| Insulin | 51.5 ± 10.8 | 28.5 ± 4.2 | 8.10 ± 1.30 | NA | 70 | 12 |
| Semaglutide | 57.8 ± 9.9 | 32.6 ± 6.4 | 8.50 ± 1.10 | 8.8 ± 5.8 | NA | 52 |
| Liraglutide | 67.2 ± 9.0 | 26.4 ± 4.6 | 8.04 ± 0.75 | NA | 70 | 24 |
| Loxenatide | 56.0 ± 9.3 | 22.1 ± 1.0 | 8.70 ± 1.70 | 9.6 ± 6.6 | 60 | 16 |
| Placebo | 59.0 ± 10.8 | 29.6 ± 4.9 | 7.89 ± 0.90 | 10.0 ± 7.3 | 54.8 | 34.8 |
| **Pooled average** | **57.9 ± 10.4** | **29.3 ± 4.9** | **7.85 ± 0.96** | **8.5 ± 6.3** | **55.2** | **41.3** |

Table S8.2: Transitivity (indirectness) assessment: geographic distribution

| **Intervention** | **Multinational (%)** | **East Asia (%)** | **Europe (%)** | **Middle East (%)** | **Latin America (%)** |
| --- | --- | --- | --- | --- | --- |
| Canagliflozin | 98.7 | 1.3 | 0 | 0 | 0 |
| Dapagliflozin | 22.1 | 63.3 | 3.7 | 0 | 10.9 |
| Empagliflozin | 0 | 54.6 | 14.8 | 30.6 | 0 |
| Ipragliflozin | 0 | 100 | 0 | 0 | 0 |
| Tofogliflozin | 0 | 100 | 0 | 0 | 0 |
| Glimepiride | 95.7 | 4.3 | 0 | 0 | 0 |
| Placebo | 44 | 40.1 | 9.1 | 6.8 | 0 |
| Pioglitazone | 0 | 0 | 0 | 100 | 0 |
| Sitagliptin | 0 | 100 | 0 | 0 | 0 |
| Metformin | 0 | 100 | 0 | 0 | 0 |
| Semaglutide | 100 | 0 | 0 | 0 | 0 |
| Liraglutide | 0 | 100 | 0 | 0 | 0 |
| Glibenclamide | 0 | 0 | 0 | 0 | 100 |
| Linagliptin | 0 | 100 | 0 | 0 | 0 |
| Insulin | 0 | 100 | 0 | 0 | 0 |
| Loxenatide | 0 | 100 | 0 | 0 | 0 |
| **Overall proportion** | **71.5** | **23.3** | **1.6** | **2.2** | **1.4** |

Table S8.3: Transitivity (indirectness) assessment: measurement methods for SMM

| **Intervention** | **Measurement method (n)** |
| --- | --- |
| Dapagliflozin | BIA (7) |
| Empagliflozin | BIA (3) |
| Ipragliflozin | BIA (1) |
| Canagliflozin | BIA (2), DXA (1) |
| Placebo | BIA (8) |
| Linagliptin | BIA (1) |
| Loxenatide | DXA (1) |

Table S8.4: Transitivity (indirectness) assessment: measurement methods for LM

| **Intervention** | **Measurement method (n)** |
| --- | --- |
| Canagliflozin | DXA (5) |
| Dapagliflozin | DXA (6) |
| Empagliflozin | DXA (3), BIA (2) |
| Ipragliflozin | DXA (2), BIA (1) |
| Tofogliflozin | BIA (1) |
| Glimepiride | DXA (3), BIA (1) |
| Placebo | DXA (9), BIA (2) |
| Sitagliptin | BIA (1) |
| Semaglutide | DXA (1) |
| Liraglutide | DXA (1) |
| Glibenclamide | DXA (1) |
| Insulin | DXA (1) |

Table S8.5: CINeMA Results of SMM

| Comparison | Within-study bias | Reporting bias | Indirectness | Imprecision | Heterogeneity | Incoherence | Confidence rating |
| --- | --- | --- | --- | --- | --- | --- | --- |
| Canagliflozin:Loxenatide | Some concerns | Low risk | No concerns | Major concerns | No concerns | No concerns | Moderate |
| Canagliflozin:Placebo | Some concerns | Low risk | No concerns | Major concerns | No concerns | No concerns | Moderate |
| Dapagliflozin:Placebo | Some concerns | Low risk | No concerns | Major concerns | No concerns | No concerns | Moderate |
| Empagliflozin:Linagliptin | Some concerns | Low risk | No concerns | Major concerns | No concerns | No concerns | Moderate |
| Empagliflozin:Placebo | No concerns | Low risk | No concerns | Major concerns | No concerns | No concerns | Moderate |
| Ipragliflozin:Placebo | Some concerns | Low risk | No concerns | Major concerns | No concerns | No concerns | Moderate |
| Canagliflozin:Dapagliflozin | Some concerns | Low risk | No concerns | Major concerns | No concerns | No concerns | Moderate |
| Canagliflozin:Empagliflozin | No concerns | Low risk | No concerns | Major concerns | No concerns | No concerns | Moderate |
| Canagliflozin:Ipragliflozin | Some concerns | Low risk | No concerns | Major concerns | No concerns | No concerns | Moderate |
| Canagliflozin:Linagliptin | Some concerns | Low risk | No concerns | Major concerns | No concerns | No concerns | Moderate |
| Dapagliflozin:Empagliflozin | No concerns | Low risk | No concerns | Major concerns | No concerns | No concerns | Moderate |
| Dapagliflozin:Ipragliflozin | Some concerns | Low risk | No concerns | Major concerns | No concerns | No concerns | Moderate |
| Dapagliflozin:Linagliptin | Some concerns | Low risk | No concerns | Major concerns | No concerns | No concerns | Moderate |
| Dapagliflozin:Loxenatide | Some concerns | Low risk | No concerns | Major concerns | No concerns | No concerns | Moderate |
| Empagliflozin:Ipragliflozin | No concerns | Low risk | No concerns | Major concerns | No concerns | No concerns | Moderate |
| Empagliflozin:Loxenatide | Some concerns | Low risk | No concerns | Major concerns | No concerns | No concerns | Moderate |
| Ipragliflozin:Linagliptin | Some concerns | Low risk | No concerns | Major concerns | No concerns | No concerns | Moderate |
| Ipragliflozin:Loxenatide | Some concerns | Low risk | No concerns | Major concerns | No concerns | No concerns | Moderate |
| Linagliptin:Loxenatide | Some concerns | Low risk | No concerns | Major concerns | No concerns | No concerns | Moderate |
| Linagliptin:Placebo | No concerns | Low risk | No concerns | Major concerns | No concerns | No concerns | Moderate |
| Loxenatide:Placebo | Some concerns | Low risk | No concerns | Major concerns | No concerns | No concerns | Moderate |

Table S8.6: CINeMA Results of LM

| Comparison | Within-study bias | Reporting bias | Indirectness | Imprecision | Heterogeneity | Incoherence | Confidence rating |
| --- | --- | --- | --- | --- | --- | --- | --- |
| Canagliflozin:Glimepiride | No concerns | Low risk | No concerns | No concerns | Major concerns | No concerns | Moderate |
| Canagliflozin:Placebo | No concerns | Low risk | No concerns | Major concerns | No concerns | No concerns | Moderate |
| Canagliflozin:Semaglutide | No concerns | Low risk | No concerns | Major concerns | No concerns | Major concerns | Low |
| Dapagliflozin:Glibenclamide | Some concerns | Low risk | No concerns | No concerns | Major concerns | Major concerns | Moderate |
| Dapagliflozin:Glimepiride | Some concerns | Low risk | No concerns | Major concerns | No concerns | No concerns | Moderate |
| Dapagliflozin:Insulin | Some concerns | Low risk | No concerns | Major concerns | No concerns | Major concerns | Low |
| Dapagliflozin:Placebo | Some concerns | Low risk | No concerns | Major concerns | No concerns | No concerns | Moderate |
| Empagliflozin:Liraglutide | Some concerns | Low risk | No concerns | Major concerns | No concerns | Major concerns | Low |
| Empagliflozin:Placebo | No concerns | Low risk | No concerns | No concerns | Major concerns | Major concerns | Moderate |
| Glimepiride:Tofogliflozin | Some concerns | Low risk | No concerns | No concerns | Major concerns | Major concerns | Moderate |
| Ipragliflozin:Placebo | Some concerns | Low risk | No concerns | No concerns | Major concerns | Major concerns | Moderate |
| Ipragliflozin:Sitagliptin | Some concerns | Low risk | No concerns | Major concerns | No concerns | Major concerns | Low |
| Canagliflozin:Dapagliflozin | No concerns | Low risk | No concerns | Major concerns | No concerns | Major concerns | Low |
| Canagliflozin:Empagliflozin | No concerns | Low risk | No concerns | Major concerns | No concerns | Major concerns | Low |
| Canagliflozin:Glibenclamide | Some concerns | Low risk | No concerns | No concerns | Major concerns | Major concerns | Moderate |
| Canagliflozin:Insulin | Some concerns | Low risk | No concerns | Major concerns | No concerns | Major concerns | Low |
| Canagliflozin:Ipragliflozin | Some concerns | Low risk | No concerns | Major concerns | No concerns | Major concerns | Low |
| Canagliflozin:Liraglutide | No concerns | Low risk | No concerns | Major concerns | No concerns | Major concerns | Low |
| Canagliflozin:Sitagliptin | Some concerns | Low risk | No concerns | Major concerns | No concerns | Major concerns | Low |
| Canagliflozin:Tofogliflozin | Some concerns | Low risk | No concerns | Major concerns | No concerns | Major concerns | Low |
| Dapagliflozin:Empagliflozin | No concerns | Low risk | No concerns | Major concerns | No concerns | Major concerns | Low |
| Dapagliflozin:Ipragliflozin | Some concerns | Low risk | No concerns | No concerns | Major concerns | Major concerns | Moderate |
| Dapagliflozin:Liraglutide | Some concerns | Low risk | No concerns | Major concerns | No concerns | Major concerns | Low |
| Dapagliflozin:Semaglutide | No concerns | Low risk | No concerns | Major concerns | No concerns | Major concerns | Low |
| Dapagliflozin:Sitagliptin | Some concerns | Low risk | No concerns | Major concerns | No concerns | Major concerns | Low |
| Dapagliflozin:Tofogliflozin | Some concerns | Low risk | No concerns | Major concerns | No concerns | Major concerns | Low |
| Empagliflozin:Glibenclamide | Some concerns | Low risk | No concerns | No concerns | Major concerns | Major concerns | Moderate |
| Empagliflozin:Glimepiride | No concerns | Low risk | No concerns | No concerns | Major concerns | Major concerns | Moderate |
| Empagliflozin:Insulin | Some concerns | Low risk | No concerns | Major concerns | No concerns | Major concerns | Low |
| Empagliflozin:Ipragliflozin | No concerns | Low risk | No concerns | Major concerns | No concerns | Major concerns | Low |
| Empagliflozin:Semaglutide | No concerns | Low risk | No concerns | Major concerns | No concerns | Major concerns | Low |
| Empagliflozin:Sitagliptin | Some concerns | Low risk | No concerns | Major concerns | No concerns | Major concerns | Low |
| Empagliflozin:Tofogliflozin | No concerns | Low risk | No concerns | Major concerns | No concerns | Major concerns | Low |
| Glibenclamide:Glimepiride | Some concerns | Low risk | No concerns | Major concerns | No concerns | Major concerns | Low |
| Glibenclamide:Insulin | Some concerns | Low risk | No concerns | Major concerns | No concerns | Major concerns | Low |
| Glibenclamide:Ipragliflozin | Some concerns | Low risk | No concerns | No concerns | No concerns | Major concerns | Moderate |
| Glibenclamide:Liraglutide | Some concerns | Low risk | No concerns | Major concerns | No concerns | Major concerns | Low |
| Glibenclamide:Placebo | Some concerns | Low risk | No concerns | Major concerns | No concerns | Major concerns | Low |
| Glibenclamide:Semaglutide | No concerns | Low risk | No concerns | Major concerns | No concerns | Major concerns | Low |
| Glibenclamide:Sitagliptin | Some concerns | Low risk | No concerns | No concerns | No concerns | Major concerns | Moderate |
| Glibenclamide:Tofogliflozin | Some concerns | Low risk | No concerns | No concerns | Major concerns | Major concerns | Moderate |
| Glimepiride:Insulin | Some concerns | Low risk | No concerns | Major concerns | No concerns | Major concerns | Low |
| Glimepiride:Ipragliflozin | Some concerns | Low risk | No concerns | No concerns | No concerns | Major concerns | Moderate |
| Glimepiride:Liraglutide | No concerns | Low risk | No concerns | Major concerns | No concerns | Major concerns | Low |
| Glimepiride:Placebo | No concerns | Low risk | No concerns | Major concerns | No concerns | Major concerns | Low |
| Glimepiride:Semaglutide | No concerns | Low risk | No concerns | Major concerns | No concerns | Major concerns | Low |
| Glimepiride:Sitagliptin | Some concerns | Low risk | No concerns | No concerns | Major concerns | Major concerns | Moderate |
| Insulin:Ipragliflozin | Some concerns | Low risk | No concerns | Major concerns | No concerns | Major concerns | Low |
| Insulin:Liraglutide | Some concerns | Low risk | No concerns | Major concerns | No concerns | Major concerns | Low |
| Insulin:Placebo | Some concerns | Low risk | No concerns | Major concerns | No concerns | Major concerns | Low |
| Insulin:Semaglutide | No concerns | Low risk | No concerns | Major concerns | No concerns | Major concerns | Low |
| Insulin:Sitagliptin | Some concerns | Low risk | No concerns | Major concerns | No concerns | Major concerns | Low |
| Insulin:Tofogliflozin | Some concerns | Low risk | No concerns | Major concerns | No concerns | Major concerns | Low |
| Ipragliflozin:Liraglutide | Some concerns | Low risk | No concerns | Major concerns | No concerns | Major concerns | Low |
| Ipragliflozin:Semaglutide | No concerns | Low risk | No concerns | Major concerns | No concerns | Major concerns | Low |
| Ipragliflozin:Tofogliflozin | Some concerns | Low risk | No concerns | Major concerns | No concerns | Major concerns | Low |
| Liraglutide:Placebo | No concerns | Low risk | No concerns | Major concerns | No concerns | Major concerns | Low |
| Liraglutide:Semaglutide | No concerns | Low risk | No concerns | Major concerns | No concerns | Major concerns | Low |
| Liraglutide:Sitagliptin | Some concerns | Low risk | No concerns | Major concerns | No concerns | Major concerns | Low |
| Liraglutide:Tofogliflozin | Some concerns | Low risk | No concerns | Major concerns | No concerns | Major concerns | Low |
| Placebo:Semaglutide | No concerns | Low risk | No concerns | Major concerns | No concerns | Major concerns | Low |
| Placebo:Sitagliptin | Some concerns | Low risk | No concerns | No concerns | Major concerns | Major concerns | Moderate |
| Placebo:Tofogliflozin | Some concerns | Low risk | No concerns | Major concerns | No concerns | Major concerns | Low |
| Semaglutide:Sitagliptin | Some concerns | Low risk | No concerns | Major concerns | No concerns | Major concerns | Low |
| Semaglutide:Tofogliflozin | No concerns | Low risk | No concerns | Major concerns | No concerns | Major concerns | Low |
| Sitagliptin:Tofogliflozin | Some concerns | Low risk | No concerns | Major concerns | No concerns | Major concerns | Low |

Figure S8.5: Contribution matrix for the SMM network

Abbreviations: CAN, canagliflozin; DAP, dapagliflozin; EMP, empagliflozin; IPRA, ipragliflozin; LIN, linagliptin; LOX, loxenatide; PBO, placebo.

Figure S8.6: Contribution matrix for the LM network

Abbreviations: CAN, canagliflozin; DAP, dapagliflozin; EMP, empagliflozin; GLIB, glibenclamide; GLIM, glimepiride; INS, insulin; IPRA, ipragliflozin; LIRA, liraglutide; SEM, semaglutide; SITA, sitagliptin; TOFO, tofogliflozin; PBO, placebo

**Appendix 9: Funnel plots**

These funnel plots are constructed to evaluate small-study effects and potential publication bias across various outcomes in the network meta-analysis of SGLT2 inhibitors. Each data point represents a direct comparison from eligible trials.

**Figure S9.1:** Funnel plot of SMM

Egger’s test t=1.07  P=0.308

**Figure S9.2:** Funnel plot of LM

Egger’s test t= - 0.83 P= 0.417

**Figure S9.3:** Funnel plot of FM

Egger’s test t= -0.26 P= 0.798

**Figure S9.4:** Funnel plot of PBF

Egger’s test t= 0.30 P= 0.770

**Figure S9.5:** Funnel plot of VFA

Egger’s test t= -4.51 P= 0.001

**Figure S9.6:** Funnel plot of SFA

Egger’s test t= 5.26  P= 0.001

**Figure S9.7:** Funnel plot of BW

Egger’s test t= - 0.10 P= 0.919

**Figure S9.8:** Funnel plot of BMI

Egger’s test t= 0.05 P= 0.959

**Figure S9.9:** Funnel plot of WC

Egger’s test t= -2.28 P= 0.039

**Figure S9.10:** Funnel plot of TBW

Egger’s test t= 0.70 P= 0.514

**Figure S9.11:** Funnel plot of any AE

Egger’s test t= -2.11 P= 0.045

**Figure S9.12:** Funnel plot of any serious AE

Egger’s test t= - 0.26 P= 0.801

**Figure S9.13:** Funnel plot of AE leading to discontinuation

Egger’s test t= - 0.38 P= 0.716

**Figure S9.14:** Funnel plot of Genitourinary tract infection

Egger’s test t= 1.23 P= 0.240

**Appendix 10: Sensitivity analyses**

Table S10.1:Sensitivity analyses for the primary outcomes (SMM and LM)

| Intervention | SMM | | | LM | | | |
| --- | --- | --- | --- | --- | --- | --- | --- |
|  | Main estimate | Excluding high risk | Excluding small studies | Main estimate | Excluding high risk | Excluding small studies | Low-risk studies only |
| Canagliflozin | -2.02(-15.01, 0.97) | -2.03(-15.05, 10.99) | NA | -0.80 (-1.39, -0.21) | -0.80 (-1.39, -0.21) | -0.80 (-1.39, -0.21) | -0.75 (-1.47,-0.03) |
| Dapagliflozin | -0.32 (-0.85, 0.21) | -0.32 (-0.85, 0.22) | -0.58 (-1.26, 0.11) | -0.13 (-0.66, 0.39) | -0.13 (-0.66, 0.39) | -0.13 (-0.66, 0.39) | -0.40 (-1.29,0.49) |
| Empagliflozin | -0.68 (-1.63, 0.26) | -0.68 (-1.63, 0.26) | -0.68 (-1.63, 0.26) | -1.22 (-1.71, -0.72) | -1.22 (-1.71, -0.72) | -1.22 (-1.71, -0.72) | -1.22 (-1.78,-0.67) |
| Glibenclamide | NA | NA | NA | 1.14 (0.32, 1.96) | 1.14 (0.32, 1.96) | 1.14 (0.32, 1.96) | NA |
| Glimepiride | NA | NA | NA | 1.25 (0.54, 1.97) | 1.25 (0.54, 1.97) | 1.25 (0.54, 1.97) | 1.35 (0.32,2.37) |
| Insulin | NA | NA | NA | 0.07 (-2.40, 2.54) | 0.07 (-2.40, 2.54) | 0.07 (-2.40, 2.54) | NA |
| Ipragliflozin | -0.59 (-5.98, 4.80) | -0.59 (-5.98, 4.80) | -0.59 (-5.98, 4.80) | -0.99 (-1.45, -0.54) | -0.99 (-1.45, -0.54) | -0.99 (-1.45, -0.54) | NA |
| Linagliptin | 0.39 (-1.05, 1.83) | 0.39 (-1.05, 1.83) | 0.39 (-1.05, 1.83) | NA | NA | NA | NA |
| Liraglutide | NA | NA | NA | -1.12 (-2.29, 0.06) | -1.12 (-2.29, 0.06) | -1.12 (-2.29, 0.06) | NA |
| Loxenatide | -2.02(-15.69, 1.65) | -2.03 (-15.72, 1.67) | NA | NA | NA | NA | NA |
| Pioglitazone | NA | NA | NA | NA | NA | NA | NA |
| Semaglutide | NA | NA | NA | -1.58 (-2.66, -0.50) | -1.58 (-2.66, -0.50) | -1.58 (-2.66, -0.50) | -1.53 (-2.77,-0.29) |
| Sitagliptin | NA | NA | NA | -0.69 (-4.11, 2.72) | -0.69 (-4.11, 2.72) | -0.69 (-4.11, 2.72) | NA |
| Tofogliflozin | NA | NA | NA | -0.95 (-2.14, 0.24) | -0.95 (-2.14, 0.24) | -0.95 (-2.14, 0.24) | NA |

**Note:** A low-risk-only sensitivity analysis was performed for LM only. For SMM, only three studies were rated as low risk of bias; therefore, a network meta-analysis restricted to low-risk studies was not feasible.

Table S10.2:BIA-only sensitivity analysis for SMM

| Intervention | SMM | |
| --- | --- | --- |
|  | Main estimate | BIA-only |
| Canagliflozin | -2.02 (-15.01,10.97) | -2.02 (-15.01,10.97) |
| Dapagliflozin | -0.32 (-0.85,0.21) | -0.32 (-0.85,0.21) |
| Empagliflozin | -0.68 (-1.63,0.26) | -0.68 (-1.63,0.26) |
| Ipragliflozin | -0.59 (-5.98,4.80) | -0.59 (-5.98,4.80) |
| Linagliptin | 0.39 (-1.05,1.83) | 0.39 (-1.05,1.83) |
| Loxenatide | -2.02 (-15.69,11.65) | NA |

Table S10.3: DXA-only sensitivity analysis for LM

| Intervention | LM | |
| --- | --- | --- |
|  | Main estimate | DXA-only |
| Canagliflozin | -0.80 (-1.39,-0.21) | -0.80 (-1.32,-0.28) |
| Dapagliflozin | -0.13 (-0.66,0.39) | -0.14 (-0.60,0.32) |
| Empagliflozin | -1.22 (-1.71,-0.72) | -1.56 (-2.10,-1.02) |
| Glibenclamide | 1.14 (0.32,1.96) | 1.14 (0.48,1.79) |
| Glimepiride | 1.25 (0.54,1.97) | 1.25 (0.61,1.89) |
| Insulin | 0.07 (-2.40,2.54) | 0.06 (-2.35,2.48) |
| Ipragliflozin | -0.99 (-1.45,-0.54) | -1.00 (-1.19,-0.81) |
| Liraglutide | -1.12 (-2.29,0.06) | -1.46 (-2.58,-0.34) |
| Semaglutide | -1.58 (-2.66,-0.50) | -1.58 (-2.54,-0.62) |
| Sitagliptin | -0.69 (-4.11,2.72) | NA |
| Tofogliflozin | -0.95 (-2.14,0.24) | NA |

Table S10.4. Sensitivity analysis for VFA excluding the study with imputed standard deviations

| Intervention | VFA | |
| --- | --- | --- |
|  | Main estimate | Excluding the study with imputed SDs |
| Dapagliflozin | -11.97 (-20.90,-3.04) | -11.97 (-20.90,-3.04) |
| Empagliflozin | -12.20 (-35.82,11.42) | -12.20 (-35.82,11.42) |
| Glimepiride | 5.57 (-11.99,23.13) | 5.62 (-11.95,23.18) |
| Ipragliflozin | -33.08 (-37.18,-28.97) | -33.08 (-37.19,-28.98) |
| Metformin | -10.76 (-22.79,1.28) | -11.40 (-23.98,1.19) |
| Pioglitazone | 2.10 (-21.67,25.87) | 2.10 (-21.66,25.87) |
| Sitagliptin | -24.58 (-38.07,-11.08) | -24.58 (-38.08,-11.09) |

Table S10.5. Sensitivity analysis for SFA excluding the study with imputed standard deviations

| Intervention | SFA | |
| --- | --- | --- |
|  | Main estimate | Excluding the study with imputed SDs |
| Dapagliflozin | -35.02 (-53.69,-16.34) | -35.26 (-54.02,-16.51) |
| Glimepiride | -16.63 (-38.78,5.53) | -16.87 (-39.10,5.35) |
| Ipragliflozin | -7.97 (-11.61,-4.33) | -7.96 (-11.60,-4.32) |
| Metformin | 12.17 (1.62,22.72) | 13.42 (1.36,25.49) |

**Appendix 11: Meta-regression analyses**

| Factors | SMM | | | LM | | |
| --- | --- | --- | --- | --- | --- | --- |
|  | Coefficient | 95% confidence interval | P value | Coefficient | 95% confidence interval | P value |
| Measurement method | 0.96 | −2.36, 4.28 | 0.540 | 0.25 | −0.90, 1.40 | 0.652 |
| Follow-up duration (≥24 vs <24 weeks) | 1.29 | -0.67, 3.24 | 0.175 | 0.26 | −0.71, 1.22 | 0.581 |

**Appendix 12**: **PRISMA-NMA Checklist**

**PRISMA NMA Checklist of Items to Include When Reporting A Systematic Review Involving a Network Meta-analysis**

| **Section/Topic** | **Item #** | **Checklist Item** | **Reported on Page #** |
| --- | --- | --- | --- |
| **TITLE** |  |  |  |
| Title | 1 | Identify the report as a systematic review *incorporating a network meta-analysis (or related form of meta-analysis).* | 1 |
|  |  |  |  |
| **ABSTRACT** |  |  |  |
| Structured summary | 2 | Provide a structured summary including, as applicable:  **Background:** main objectives  **Methods:** data sources; study eligibility criteria, participants, and interventions; study appraisal; and *synthesis methods, such as network meta-analysis.*  **Results:** number of studies and participants identified; summary estimates with corresponding confidence/credible intervals; *treatment rankings may also be discussed. Authors may choose to summarize pairwise comparisons against a chosen treatment included in their analyses for brevity.*  **Discussion/Conclusions:** limitations; conclusions and implications of findings.  **Other:** primary source of funding; systematic review registration number with registry name. | 1-2 |
|  |  |  |  |
| **INTRODUCTION** |  |  |  |
| Rationale | 3 | Describe the rationale for the review in the context of what is already known*, including mention of why a network meta-analysis has been conducted.* | 2-3 |
| Objectives | 4 | Provide an explicit statement of questions being addressed, with reference to participants, interventions, comparisons, outcomes, and study design (PICOS). | 3 |
|  |  |  |  |
| **METHODS** |  |  |  |
| Protocol and registration | 5 | Indicate whether a review protocol exists and if and where it can be accessed (e.g., Web address); and, if available, provide registration information, including registration number. | 3 |
| Eligibility criteria | 6 | Specify study characteristics (e.g., PICOS, length of follow-up) and report characteristics (e.g., years considered, language, publication status) used as criteria for eligibility, giving rationale. *Clearly describe eligible treatments included in the treatment network, and note whether any have been clustered or merged into the same node (with justification).* | 4 |
| Information sources | 7 | Describe all information sources (e.g., databases with dates of coverage, contact with study authors to identify additional studies) in the search and date last searched. | 3-4 |
| Search | 8 | Present full electronic search strategy for at least one database, including any limits used, such that it could be repeated. | Appendix 1 |
| Study selection | 9 | State the process for selecting studies (i.e., screening, eligibility, included in systematic review, and, if applicable, included in the meta-analysis). | 4, 7–8 |
| Data collection process | 10 | Describe method of data extraction from reports (e.g., piloted forms, independently, in duplicate) and any processes for obtaining and confirming data from investigators. | 5 |
| Data items | 11 | List and define all variables for which data were sought (e.g., PICOS, funding sources) and any assumptions and simplifications made. | 5 |
| **Geometry of the network** | **S1** | Describe methods used to explore the geometry of the treatment network under study and potential biases related to it. This should include how the evidence base has been graphically summarized for presentation, and what characteristics were compiled and used to describe the evidence base to readers. | 7 |
| Risk of bias within individual studies | 12 | Describe methods used for assessing risk of bias of individual studies (including specification of whether this was done at the study or outcome level), and how this information is to be used in any data synthesis. | 5-6 |
| Summary measures | 13 | State the principal summary measures (e.g., risk ratio, difference in means). *Also describe the use of additional summary measures assessed, such as treatment rankings and surface under the cumulative ranking curve (SUCRA) values, as well as modified approaches used to present summary findings from meta-analyses.* | 6-7 |
| Planned methods of analysis | 14 | Describe the methods of handling data and combining results of studies for each network meta-analysis. This should include, but not be limited to:   - *Handling of multi-arm trials;* - *Selection of variance structure;* - *Selection of prior distributions in Bayesian analyses; and* - *Assessment of model fit.* | 6-7 |
| **Assessment of Inconsistency** | **S2** | Describe the statistical methods used to evaluate the agreement of direct and indirect evidence in the treatment network(s) studied. Describe efforts taken to address its presence when found. | 6 |
| Risk of bias across studies | 15 | Specify any assessment of risk of bias that may affect the cumulative evidence (e.g., publication bias, selective reporting within studies). | 7 |
| Additional analyses | 16 | Describe methods of additional analyses if done, indicating which were pre-specified. This may include, but not be limited to, the following:   - Sensitivity or subgroup analyses; - Meta-regression analyses; - *Alternative formulations of the treatment network; and* - *Use of alternative prior distributions for Bayesian analyses (if applicable).* | 6–7; Appendix 10–11 |
|  |  |  |  |
| **RESULTS†** |  |  |  |
| Study selection | 17 | Give numbers of studies screened, assessed for eligibility, and included in the review, with reasons for exclusions at each stage, ideally with a flow diagram. | 7–8 |
| **Presentation of network structure** | **S3** | Provide a network graph of the included studies to enable visualization of the geometry of the treatment network. | 10–13; Appendix 5 |
| **Summary of network geometry** | **S4** | Provide a brief overview of characteristics of the treatment network. This may include commentary on the abundance of trials and randomized patients for the different interventions and pairwise comparisons in the network, gaps of evidence in the treatment network, and potential biases reflected by the network structure. | 10–13; Appendix 5 |
| Study characteristics | 18 | For each study, present characteristics for which data were extracted (e.g., study size, PICOS, follow-up period) and provide the citations. | 7–8; Appendix 2 |
| Risk of bias within studies | 19 | Present data on risk of bias of each study and, if available, any outcome level assessment. | 8–9; Appendix 3 |
| Results of individual studies | 20 | For all outcomes considered (benefits or harms), present, for each study: 1) simple summary data for each intervention group, and 2) effect estimates and confidence intervals. *Modified approaches may be needed to deal with information from larger networks.* | Appendix 2; Appendix 7 |
| Synthesis of results | 21 | Present results of each meta-analysis done, including confidence/credible intervals. *In larger networks, authors may focus on comparisons versus a particular comparator (e.g. placebo or standard care), with full findings presented in an appendix. League tables and forest plots may be considered to summarize pairwise comparisons.* If additional summary measures were explored (such as treatment rankings), these should also be presented. | 10–20; Appendix 5–7 |
| **Exploration for inconsistency** | **S5** | Describe results from investigations of inconsistency. This may include such information as measures of model fit to compare consistency and inconsistency models, *P* values from statistical tests, or summary of inconsistency estimates from different parts of the treatment network. | 9;  Appendix 4 |
| Risk of bias across studies | 22 | Present results of any assessment of risk of bias across studies for the evidence base being studied. | 9;  Appendix 9 |
| Results of additional analyses | 23 | Give results of additional analyses, if done (e.g., sensitivity or subgroup analyses, meta-regression analyses*, alternative network geometries studied, alternative choice of prior distributions for Bayesian analyses,* and so forth). | Appendix 10–11 |
|  |  |  |  |
| **DISCUSSION** |  |  |  |
| Summary of evidence | 24 | Summarize the main findings, including the strength of evidence for each main outcome; consider their relevance to key groups (e.g., healthcare providers, users, and policy-makers). | 20–22; Appendix 8 |
| Limitations | 25 | Discuss limitations at study and outcome level (e.g., risk of bias), and at review level (e.g., incomplete retrieval of identified research, reporting bias). *Comment on the validity of the assumptions, such as transitivity and consistency. Comment on any concerns regarding network geometry (e.g., avoidance of certain comparisons).* | 22–23 |
| Conclusions | 26 | Provide a general interpretation of the results in the context of other evidence, and implications for future research. | 23 |
|  |  |  |  |
| **FUNDING** |  |  |  |
| Funding | 27 | Describe sources of funding for the systematic review and other support (e.g., supply of data); role of funders for the systematic review. This should also include information regarding whether funding has been received from manufacturers of treatments in the network and/or whether some of the authors are content experts with professional conflicts of interest that could affect use of treatments in the network. | 23 |

PICOS = population, intervention, comparators, outcomes, study design.

* Text in italics indicateS wording specific to reporting of network meta-analyses that has been added to guidance from the PRISMA statement.

† Authors may wish to plan for use of appendices to present all relevant information in full detail for items in this section.
